# A new desert-dwelling dinosaur (Theropoda, Noasaurinae) from the Cretaceous of south Brazil

**DOI:** 10.1038/s41598-019-45306-9

**Published:** 2019-06-26

**Authors:** Max Cardoso Langer, Neurides de Oliveira Martins, Paulo César Manzig, Gabriel de Souza Ferreira, Júlio César de Almeida Marsola, Edison Fortes, Rosana Lima, Lucas Cesar Frediani Sant’ana, Luciano da Silva Vidal, Rosangela Honório da Silva Lorençato, Martín Daniel Ezcurra

**Affiliations:** 1 Laboratório de Paleontologia, Faculdade de Filosofia, Ciências e Letras de Ribeirão Preto, Universidade de São Paulo, Av. Bandeirantes, 3900, 14040-901 Ribeirão Preto/SP, Brazil; 2 Museu de Paleontologia de Cruzeiro do Oeste, Rua João Ormino de Rezende, 686, 87400-000 Cruzeiro do Oeste/PR, Brazil; 3 Centro de Estudos Paleontologicos, Ambientais e Culturais (Cepac), Rua Edmundo Mercer Junior, 1308, 87400-000 Cruzeiro do Oeste/PR, Brazil; 4 Programa de Pós-Graduação em Geografia (PGE), Universidade Estadual de Maringá, Avenida Colombo, 5790, 87020-900 Maringá/PR, Brazil; 5 Grupo de Estudos Multidisciplinares do Ambiente (GEMA), Universidade Estadual de Maringá, Avenida Colombo, 5790, 87020-900 Maringá/PR, Brazil; 6 Laboratório de Macrofósseis, Universidade Federal do Rio de Janeiro, Av. Athos da Silveira Ramos, 274, 21941-611 Rio de Janeiro/RJ, Brazil; 7 Sección Paleontología de Vertebrados, CONICET−Museo Argentino de Ciencias Naturales “Bernardino Rivadavia”, Avenida Ángel Gallardo, 470, C1405DJR Buenos Aires, Argentina

**Keywords:** Palaeontology, Herpetology

## Abstract

Noasaurines form an enigmatic group of small-bodied predatory theropod dinosaurs known from the Late Cretaceous of Gondwana. They are relatively rare, with notable records in Argentina and Madagascar, and possible remains reported for Brazil, India, and continental Africa. In south-central Brazil, the deposits of the Bauru Basin have yielded a rich tetrapod fauna, which is concentrated in the Bauru Group. The mainly aeolian deposits of the Caiuá Group, on the contrary, bear a scarce fossil record composed only of lizards, turtles, and pterosaurs. Here, we describe the first dinosaur of the Caiuá Group, which also represents the best-preserved theropod of the entire Bauru Basin known to date. The recovered skeletal parts (vertebrae, girdles, limbs, and scarce cranial elements) show that the new taxon was just over 1 m long, with a unique anatomy among theropods. The shafts of its metatarsals II and IV are very lateromedially compressed, as are the blade-like ungual phalanges of the respective digits. This implies that the new taxon could have been functionally monodactyl, with a main central weight-bearing digit, flanked by neighbouring elements positioned very close to digit III or even held free of the ground. Such anatomical adaptation is formerly unrecorded among archosaurs, but has been previously inferred from footprints of the same stratigraphic unit that yielded the new dinosaur. A phylogenetic analysis nests the new taxon within the Noasaurinae clade, which is unresolved because of the multiple alternative positions that *Noasaurus leali* can acquire in the optimal trees. The exclusion of the latter form results in positioning the new dinosaur as the sister-taxon of the Argentinean *Velocisaurus unicus*.

## Introduction

Noasaurinae represents an enigmatic group of small-bodied predatory theropod dinosaurs best known from the iconic *Masiakasaurus knopfleri*, from the latest Cretaceous (Maastrichtian) of Madagascar^1–3^. Yet, the clade also minimally includes two other Late Cretaceous taxa from Argentina^4^, the name-bearing *Noasaurus leali*^5,6^, as well as *Velocisaurus unicus*^7,8^. In the most recent phylogenetic revision of the group^4^, Noasaurinae was found as sister to the Middle-Late Jurassic Elaphrosaurinae, together forming Noasauridae among the abelisauroid theropods. Other taxa possibly related to Noasaurinae includes a set of Cretaceous theropods from Gondwana, i.e.: *Laevisuchus indicus*^2,8–13^ and an unnamed form from India^14^, the Moroccan *Deltadromeus agilis*^11,15,16^, the Malagasy *Dahalokely tokana*^10,17^, an unnamed form from Niger^18^, and the Argentinean *Ligabueino andesi*^17^ and *Austrocheirus isasii*^17^, as well as the peri-Gondwanan *Genusaurus sisteronis* from France^10–12^. Yet, in part due to the scarce remains attributable to some of them, the relationships among these taxa and core-noasaurines are controversial and, at least for *G*. *sisteronis* and *Da*. *tokana*, there is more recent and substantial evidence^4,17–19^ suggesting that they do not belong to the Noasaurinae lineage, whereas *De*. *agilis* may instead represent a tetanuran theropod^20^.

The Bauru Basin was a sag basin developed in the south-central part of the South American Platform^21^, positioned in paleolatitudes corresponding to the “Southern Hot Arid Belt”^22^. Its deposits, i.e. the Bauru Supersequence^23^, lie upon the Valanginian-Aptian Serra Geral basalt flows^24^ and include the Caiuá and Bauru groups, which were respectively deposited in more aeolian and fluvial settings^21,25^. For some authors^21^, those stratigraphic units were chronocorrelated, respectively representing an inner desert and its surrounding alluvial environments. A more classical view^26^, endorsed by other researchers^25,27^, suggests that the Cauiá Group is older, representing a desert phase soon replaced by the more humid conditions recorded in the Bauru Group.

The fossil content of the Bauru Basin is mainly concentrated in the Bauru Group, which records a plethora of vertebrates, including dinosaurs, crocodilians, turtles, lizards, anurans, birds, and mammals^28–30^. As a whole, the fauna of the Bauru Group indicates a Coniacian to Maastrichtian age^27^, as corroborated by a recent radioisotopic dating^31^. Fossils are much rarer in the Caiuá Group, hampering the determination of its biostratigraphic correlations. These include an incomplete turtle shell from the Santo Anastácio Formation at Rubinéia-SP^32^, as well as the much richer fauna of Cruzeiro do Oeste-PR, which includes the new dinosaur described here. The Cruzeiro do Oeste fauna was first recognized by Manzig & Weinschütz^33^ and so far includes exceptionally preserved tapejarid pterosaurs, i.e. *Caiuajara dobruskii*^34^, and an acrodont lizard, i.e. *Gueragama sulamericana*^35^.

Dinosaurs are so far unknown from Caiuá Group deposits, whereas those from the Bauru Group include several titanosaur sauropods, as well as very fragmentary theropod remains^36,37^, including mostly abelisaurids^36–44^, but also megaraptorids^45,46^, unenlagiids^47^, noasaurids^48^, and improbably carcharodontosaurids^49^. Here, we describe the first dinosaur remains from the Caiuá Group, which also represent, to date, the most completely known theropod for the entire Bauru Basin. Moreover, the nesting of the new dinosaur within Noasaurinae improves our understanding of the anatomy of the group and of the evolution of some key anatomical features among abelisauroid theropods.

## Geological Settings

The Caiuá Group represents a Cretaceous quartz-sandstone sedimentary sequence^21^ deposited under arid conditions over most of the northern portions of the Serra Geral basalt flow. It has been assigned an Aptian-Albian age, based on its close association to those Valanginian-Aptian^24^ basalts and on the occurrence of tapejarid pterosaurs^25^. Nonetheless, the scarce record of pterosaurs in general casts doubts upon their utility for such correlations. On the other hand, a younger (Late Cretaceous) age can be suggested based on its inferred depositional synchronicity^21^ with rocks of the recently dated (as Coniacian to Campanian) Adamantina Formation^31^. According to some stratigraphic schemes, the Caiuá Group is composed of three coeval units: the Rio Paraná, Goio Erê, and Santo Anastácio formations. Previous accounts^34,35^ assign the fossil-bearing deposits of Cruzeiro do Oeste to the Goio Erê Formation, but the site is located in an area more commonly mapped as covered by sediments of the Rio Paraná Formation^50^. This assignment is confirmed here, based on a recent *in loco* revaluation of the stratigraphic relations and sedimentary structures present in the area.

**Figure 1:**
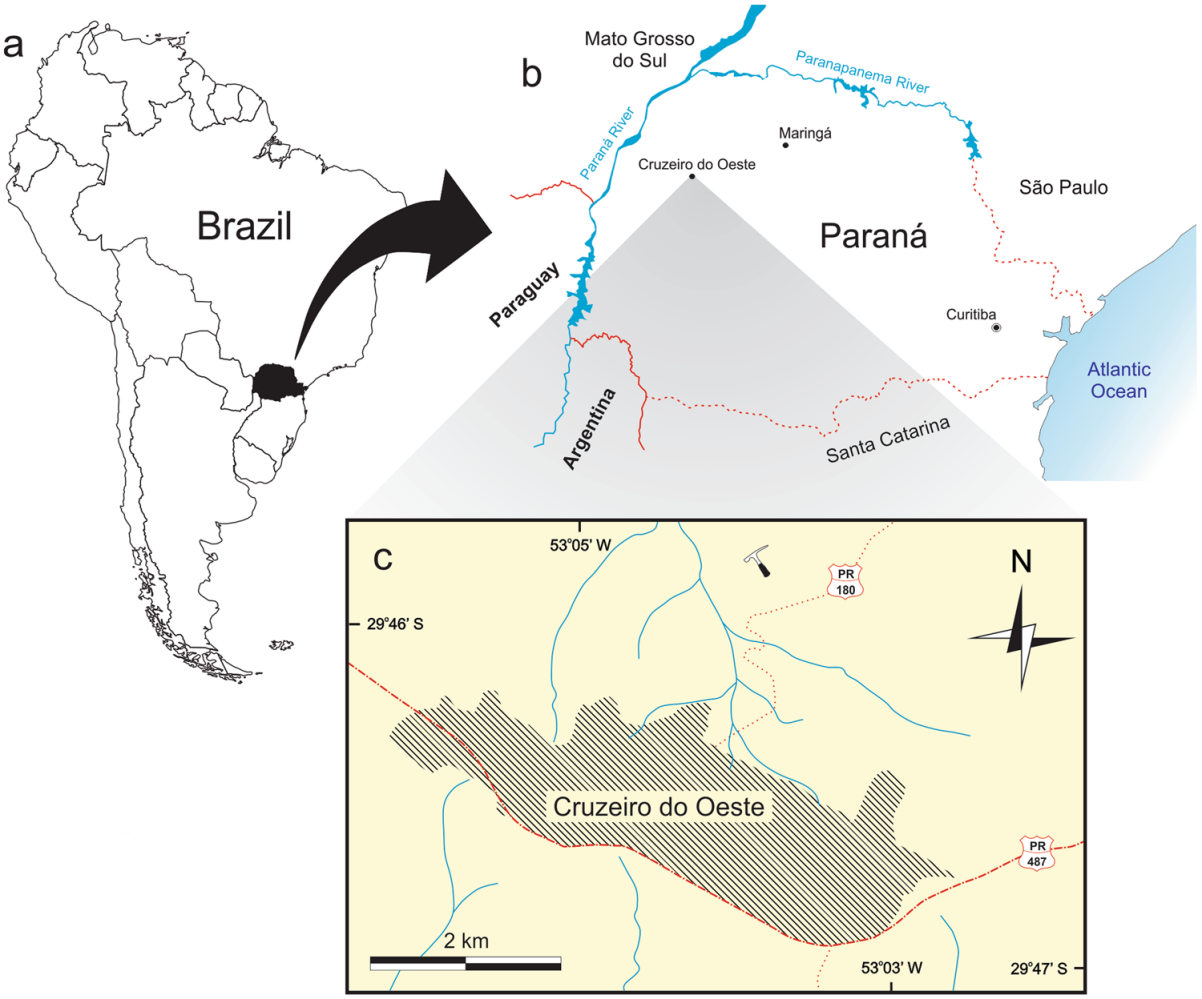
Location maps for the type-locality of *Vespersaurus paranaensis* gen. et sp. nov. (**a**) South America highlighting Paraná state (in black), Brazil. (**b**) Paraná state showing the location of Cruzeiro do Oeste. (**c**) Surroundings of the urban area (hatched) of Cruzeiro do Oeste showing the location of the type-locality (hammer). Watercourses marked in blue. PR-487, paved road; PR-180, unpaved road.

**Figure 2:**
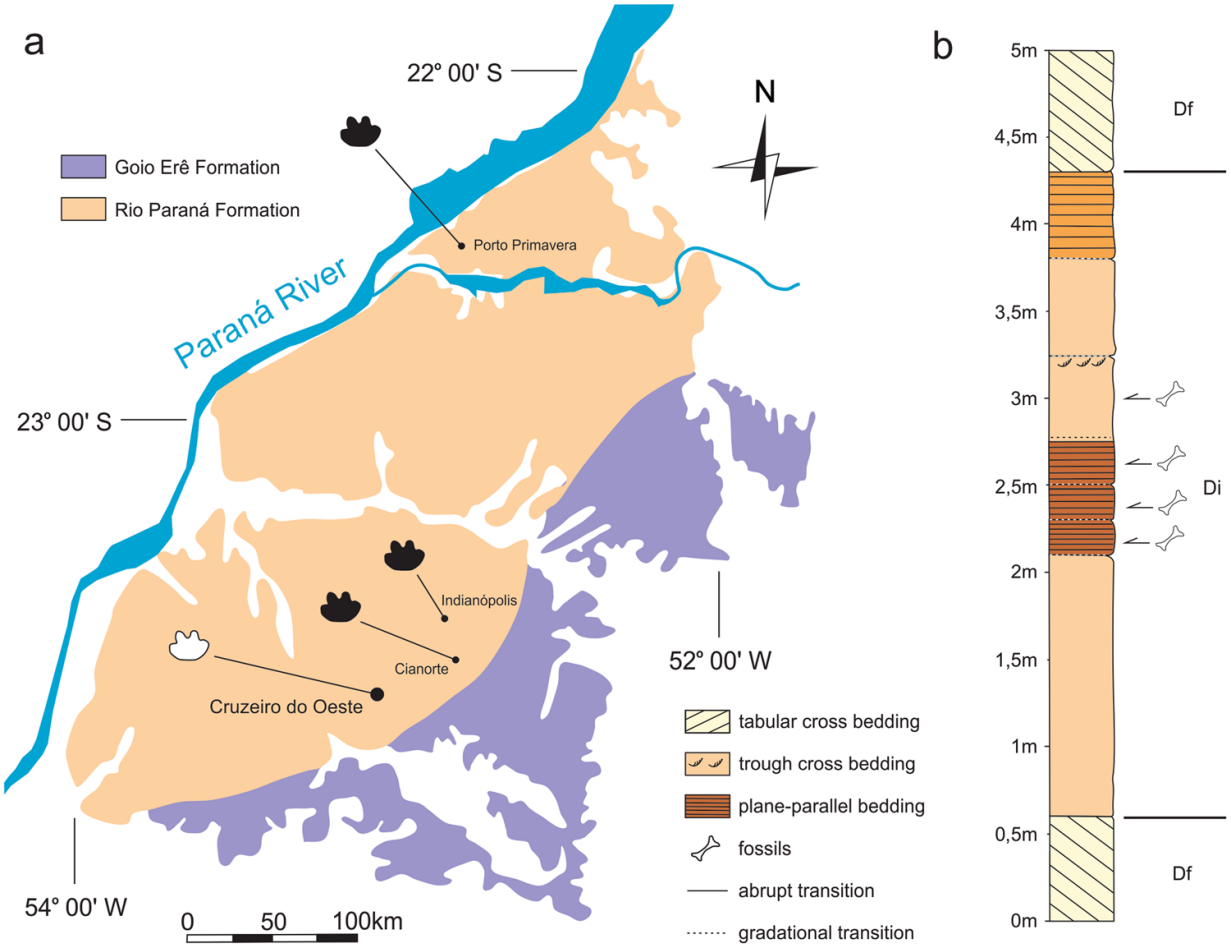
Geological context for the type-locality of *Vespersaurus paranaensis* gen. et sp. nov. (**a**) Map showing surface exposure of the Rio Paraná and Goio Erê formations in western Paraná and south-western São Paulo (modified from^21^) and the location of sites with dinosaur and mammal (black footprint) and mammal only (white footprint) tracks. (**b**) Stratigraphic column of the Rio Paraná Formation in the type-locality (indicated in Fig. 1), showing the provenance of the fossils.

In the studied area (Fig. 1), outcrops occur at hill tops and are composed of texturally and compositionally supermature, fine and very fine sandstones. These are reddish brown, with purple lamination. Three lithofacies (Fig. 2) could be identified: (1) the basalmost (Df) is composed of large cross-stratified sandstones, dipping SW (10° at the base, 25° at the top) and formed in the frontal face (lee side) of aeolian dunes, with an abrupt transition (cuneiform at the top) to the second lithofacies; (2) the second (Di) lithofacies is composed of massive reddish brown sandstones, with poorly defined plane-parallel bedding and faint centimetric ripple marks, deposited in wet interdunes, with the rich fossiliferous levels that yielded the specimen described herein, abruptly contacting the uppermost lihofacies; (3) the third (Df) lithofacies includes tangential cross stratification, dipping 15° NW, also corresponding to the frontal face of the dune. Although the depositional conditions of the Rio Paraná Formation can be described as primarily aeolian, representing a desert environment, the fossils and facies of the locality where the new dinosaur was found indicate the presence of enduring water bodies, most likely formed in interdune settings.

## Material and Methods

The phylogenetic relationships of the new species was analysed using the dataset compiled by Rauhut and Carrano^4^, which is specially focused on noasaurid interrelationships. We added two extra characters and modified some scorings of the original version of this data matrix (see Supporting Information). As per the original study^4^, we deactivated two terminal entries (*Spinostropheus gautieri* and USNM 8415) and included the new dinosaur. In a second run of the analysis, we deactivated *De*. *agilis*, because this species probably represents a tetanuran theropod rather than a ceratosaur^20^. Thus, that second run was carried out based on 31 taxa and 220 characters (see Supporting Information).

The data matrix was analysed under equally weighted maximum parsimony using TNT v.1.5^51,52^. A heuristic search of 1,000 replications of Wagner trees followed by TBR branch-swapping algorithm (holding 10 trees per replication) was performed. The best trees obtained were subjected to a final round of TBR branch swapping. Zero-length branches in any of the recovered most parsimonious trees were collapsed. Only two characters (33 and 165) were set as ordered (=additive) in the original study^4^, but we considered characters 33, 103, 104, 117, 138, 154, and 165 as additive due to their character-states representing nested sets of homologies.

Branch support was quantified using decay indices (Bremer support values^53^) and a bootstrap resampling analysis, using 1,000 pseudoreplicates and reporting both absolute and GC (that is, the difference between the frequencies of recovery in pseudoreplicates of the clade in question and the most frequently recovered contradictory clade) frequencies^51^. The minimum number of additional steps necessary to generate alternative, suboptimal tree topologies was calculated by constraining the position of terminal taxa in different parts of the tree and rerunning the analysis.

Branches or terminals that were topologically unstable through the recovered most parsimonious trees, producing polytomies in the strict consensus tree, were identified using the iterPCR protocol^54^. The iterPCR is based on an iterative evaluation of the agreement of triplets through the population of optimal trees^54^.

## Results

### Systematic Paleontology

Theropoda Marsh 1881 *sensu* Gauthier^55^

Ceratosauria Marsh, 1884 *sensu* Holtz and Padian^56^

Abelisauroidea (Bonaparte and Novas, 1985) *sensu* Wilson *et al*.^15^

Noasauridae Bonaparte & Powell, 1980 *sensu* Wilson *et al*.^15^

Noasaurinae (Bonaparte & Powell, 1980) *sensu* Rauhut and Carrano^4^

*Vespersaurus paranaensis* gen. et sp. nov. (Figs 3–12).

**Figure 3:**
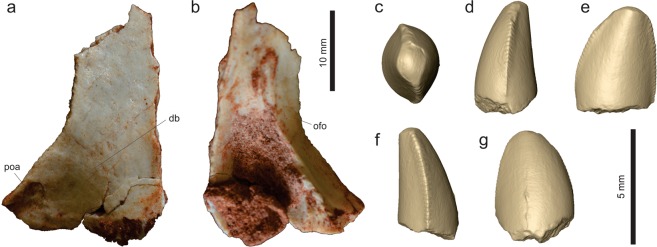
Cranial and dental remains of *Vespersaurus paranaensis* gen. et sp. nov. (**a**,**b**) Isolated frontal (MPCO.V 0063b) in dorsal (**a**) and ventral (**b**) views. (**c–g**) Isolated tooth (MPCO.V 0020c) in apical (**c**), mesial (**d**), lingual (**e**), distal (**f**), and labial (**g**) views. Anatomical abbreviations: db, dorsal bulbous area; ofo, orbital fossa; poa, postorbital articulation.

### Etymology

The generic name derives from the words “vesper” (Latin for evening/west) and “sauros” (Greek for lizard/saurian), in reference to the name of the town, i.e. Cruzeiro do Oeste (=“Western Cross”), where the fossils were found. The specific epithet refers to the Paraná state, of which *Ves*. *paranaensis* represents the first non-avian dinosaur record.

### Holotype

MPCO.V (Museu de Paleontologia de Cruzeiro do Oeste, Cruzeiro do Oeste, Brazil) 0065d, postcranial remains possibly belonging to a same individual including three trunk (MPCO.V 0065d10a-c), three sacral (MPCO.V 0065d6-8), and three tail (MPCO.V 0065d4-5,9) vertebrae, partial left ilium (MPCO.V 0065d11) and ischium (MPCO.V 0065d2), and partial articulated right pes (MPCO.V 0065d1). The assignment of those different skeletal parts to the same individual is tentative and based on the absence of duplicated elements, their close association, and matching phylogenetic signal. Numerous isolated pterosaur bones (MPCO.V 0065p) were also found, mixed with the dinosaur remains, in the same block of sediment.

### Referred material

MPCO.V 0063b, left frontal; MPCO.V 0020c, tooth; MPCO.V 0067, axis; MPCO.V 0017, 0034, 0035, 0048, postaxial neck vertebrae; MPCO.V 0010, 0040, 0062a-b, 0066, trunk vertebrae; MPCO.V 0064a, sacral vertebrae; MPCO.V 0020, 0024, 0025, 0026, 0027, 0029, 0052, 0061, tail vertebrae; MPCO.V 0011, partial right scapulocoracoid; MPCO.V 0013, partial left scapulocoracoid; MPCO.V 0006 f, left scapular fragment; MPCO.V 0006d, left humerus; MPCO.V 0006j, left radius; MPCO.V 0064b, partial articulated right hand; MPCO.V 0042, partial right pubis; MPCO.V 0014, partial left ischium; MPCO.V 0018, right tibial shaft; MPCO.V 0057b, left metatarsal I; MPCO.V 0016, third distal tarsal and partial metatarsals II and III from the left side; MPCO.V 0055, MPCO.V 0063a, two partial right metatarsals II; MPCO.V 0044, 0059, two phalanges 2 of digit III; MPCO.V 0049, 0054, two phalanges 1 of digit III; MPCO.V 0056a-b, articulated right phalanges 1–2 of digit IV; MPCO.V 0060, right phalanx 1 of digit IV; MPCO.V 0057a, left phalanx 2 of digit IV; MPCO.V 0006k, articulated left phalanges 2–3 of digit IV; MPCO.V 0022, 0036a, two pedal ungual phalanges. These elements and the holotype were found in a mixed assemblage of dinosaur and pterosaur bones, over an area of about 500 square meters, with almost no evidence of closer association between parts. They are tentatively assigned to *Ves*. *paranaensis* based on their matching size and phylogenetic signal.

### Type-locality and horizon

Dark red sandstones of the likely early Late Cretaceous Rio Paraná Formation^21,50^, exposed at the overbanks of a secondary road (53° 03′ 53″W, 26° 45′ 34″S) about 2 km north of Cruzeiro do Oeste, Paraná, Brazil (Fig. 1).

### Diagnosis

Distinguished from all other core-noasaurines (i.e. *N*. *leali*, *Mas*. *knopfleri*, and *Vel*. *unicus*) by the following unique combination of traits (unless explicitly mentioned, such differences are relative only to *Mas*. *knopfleri*) found in the holotype: ischiadic peduncle of the ilium not significantly projected ventrally relative to the pubic peduncle; ischium with no obturator notch; metatarsal II with an extremely lateromedially compressed shaft (≠ from *Vel*. *unicus*, *Mas*. *knopfleri*, and *N*. *leali*); distal articulation of metatarsal II with a shallower ventral sulcus and a lateromedialy broader medial flange (≠ from *Mas*. *knopfleri* and *N*. *leali*); lateromedially compressed metatarsal IV (≠ from *Mas*. *knopfleri*; = to *Vel*. *unicus*); pedal ungual of the fourth digit with a proximal end dorsoventrally taller than the distal end of phalanx IV-1 (≠ from *Vel*. *unicus*). In addition, other potential unique traits found in the referred material include (differences also relative only to *Mas*. *knopfleri* if not explicitly mentioned): frontal lacking a well-rimed upper temporal fossa; cranial margin of the axial diapophysis not separated from the lateral surface of the neural arch; axial epipophysis projecting distinctly beyond the postzygapophyseal facet; no cranial or caudal peduncular fossae in cranial post-axial neck vertebrae; mid-cervical vertebrae with prezygoepipophyseal lamina not cranially extensive (≠ from *N*. *leali*; = to *Mas*. *knopfleri*); mid-cervical vertebrae with epipophysis shorter than the length of the postzygapophyseal facet (≠ from *N*. *leali*; = to *Mas*. *knopfleri*); scapula blade not narrowing at its dorsal two-thirds; coracoid with a drop-shaped groove on the caudal portion of its lateral surface and a nearly straight caudal margin; humeral head more than twice (lateromedially) longer than (craniocaudally) broad in proximal view; humerus with convex distal condyles; pubis with an ambiens tuberosity on the dorsal surface of its proximal body; medial surface of the pubis with a semi-lunate depression caudal to the obturator foramen.

### Description

Based on the proportions of its holotype remains (MPCO.V 0065d), *Ves*. *paranaensis* was a small theropod, with an estimated body length of 1.0 to 1.5 meters. This is comparable in size to *N*. *leali*^5^ and the small individual (FMNH PR 2485) of *Mas*. *knopfleri*^3^, but larger than the known specimens of *Vel*. *unicus*^6^, and smaller than the adult of *Limusaurus inextricabilis*^57^. In order to estimate the body mass of *Ves*. *paranaensis* based on the equation of Campione *et al*.^58^, we employed the Benson *et al*.^59^ equation to infer femoral shaft circumference based on the tibial shaft circumference (46.18 mm in MPCO.V 0018). The resulting body mass estimate was 11.28 kg, similar to that of the ornithischians *Jeholosaurus shangyuanensis* and *Gasparinisaura cincosaltensis*.

#### Skull

An isolated left frontal (MPCO.V 0063b; Fig. 3a,b) is the single identified skull element recovered for *Ves*. *paranaensis*. It lacks its rostral third and the caudal margin is also incomplete. The bone is dorsoventrally flattened and slight arched upwards. It has a straight medial margin for the articulation with the antimere and a concave lateral margin, forming the large orbital rim, which is serrated at its central part. Although it resembles the frontal of *Mas*. *knopfleri*^3^ in general aspects, it is not a slender of an element, but nearly twice as broad (lateromedially) at its caudal margin than at its minimal breadth. The dorsal surface of the bone bears a finger-like depression at its caudolateral corner for the articulation of the postorbital. Medial to that, the bone lacks a well-rimmed upper temporal fossa similar to that of *Mas*. *knopfleri*^3^. Instead, a bulbous area (“db” in Fig. 3a) is present craniomedial to the postorbital articulation, directly in the position occupied by the cerebral hemisphere fossa within the internal surface of the bone of *Mas*. *knopfleri*^3^. That part of the ventral surface is covered by sediment in the frontal of *Ves*. *paranaensis*, but its lateral portion bears a marked orbital fossa, which maintains a similar lateromedial breadth along its preserved length and has a sharp ridge forming its medial margin.

#### Dentition

A single mostly complete tooth crown (MPCO.V 0020c) was recovered from the sample (Fig. 3c–g; see Supporting Information). It was not preserved in direct association with the holotype, but mixed with other specimens, including pterosaur material. It is short: 5 mm tall (apicobasally) and 4 mm maximally broad mesiodistally. The apex is worn down, apparently as the result of wearing during the life of the animal, resulting in a general rounded labial/lingual profile. The mesial margin is markedly convex in labial/lingual view, whereas the distal margin is straight to slightly convex. This results in a nearly straight crown long axis, subtly curved backwards. The mesial and distal carinae are well-marked, but the crown is not strongly flattened labiolingually, being more than 3 mm broad on that axis near its base. In mesial/distal views, the crown has a subtly concave lingual and a convex labial margin, resulting in a lingually curved crown. In apical view, the carinae divide the crown in equally convex lingual and labial surfaces, but the latter is slightly more extensive. The carinae are oriented somewhat lingually, so that a subtle concavity is seen flanking them on the lingual surface, resulting in a “D-shaped” cross section of the crown^60^. The distal serration extends until the base of the crown, whereas that of the mesial carina finishes about 1 mm apical to it. The denticles are of similar size and development in both carinae, with 5–6 elements per millimetre, tending to be smaller closer to the base. The tooth lacks enamel wrinkles^61,62^ and extensive interdenticular sulci^60,62–64^.

If found isolated within a sample of multiple Cretaceous theropod taxa, the affinities of MPCO.V 0020c would probably be somewhat ambiguous, but here we suggest that it belongs to the same noasaurid taxon as the other skeletal remains under description. As such, it contrasts to the highly modified rostral teeth of *Mas*. *knopfleri*^2^, or isolated *Masiakasaurus*-like teeth from the Early Cretaceous of Brazil^65^. However, when compared with other theropods (including abelisauroids^12,60,66–68^), the combination of a “D-shaped” cross section and both carinae located in the lingual part of the tooth, indicates that it belongs to the same part of the jaw, i.e. premaxilla or rostral portion of the dentary. This hampers comparisons with the more elongated and distally curved maxillary teeth of *N*. *leali* and the more blade-like “distal teeth”^66^ of *Mas*. *knopfleri*. As these come from different (more caudal) portions of the tooth series, the identified differences are more likely related to tooth position than to taxonomy. Accordingly, we infer that MPCO.V 0020c belongs to the rostral part of the jaw of a noasaurid that did not share the extreme dental modifications seen in *Mas*. *knopfleri*^1^. It is, instead, more like the supposedly plesiomorphic rostral teeth seen in other abelisauroids^66,67^.

#### Neck

The cervical part of the axial skeleton of *Ves*. *paranaensis* is represented by an axial neural arch and several isolated postaxial elements. The latter include two disarticulated centra, three disarticulated neural arches, and two elements with those parts articulated, from the cranial (3rd to 6th positions) portion of the neck, as well as one rather complete caudal (8th or 9th position) cervical vertebra.

**Figure 4:**
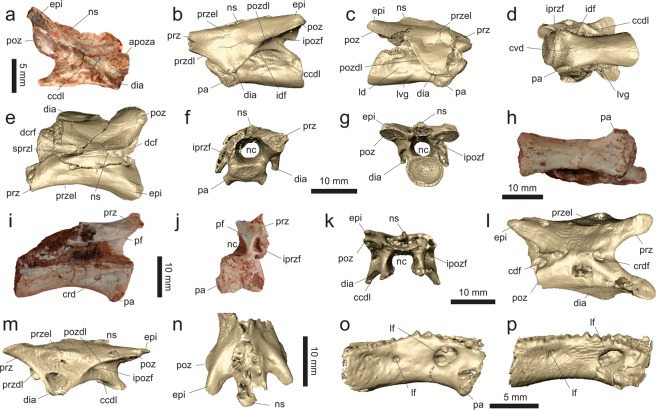
Mid-cranial neck vertebrae of *Vespersaurus paranaensis* gen. et sp. nov. (**a**) Axis (MPCO.V 0067) in right lateral view; (**b–g**) cranial cervical vertebra (MPCO.V 0035) in left (**b**) and right (**c**) lateral, ventral (**d**), dorsal (**e**), cranial (**f**), and caudal (**g**) views. (**h–j**) Cranial cervical vertebra (MPCO.V 0048) in ventral (**h**), right lateral, with a medial exposure of the left neural arch (**i**), and cranial (**j**) views. (**l**,**m**) Cranial cervical neural arch (MPCO.V 0034a) in dorsal (**l**) and left lateral (**m**) views. (**n**) Cranial cervical neural arch (MPCO.V 0034b) in dorsal view. (**o**) Cranial cervical centrum (MPCO.V 0034c) in right lateral view. (**p**) Cranial cervical centrum (MPCO.V 0034d) in right lateral view. Anatomical abbreviations: apoza, atlantal postzygapophysis articulation facet; ccdl, caudal centrodiapophyseal lamina; dcf, caudodorsal fossa; dcrf, craniodorsal fossa; cvd, cranioventral depression; dia, diapophysisi; epi, epipophysis; idf, infradiapophyseal fossa; ipozf, infrapostzygapophyseal fossa; ipref, infraprezygapophyseal fossa; ld, lateral depression; lf, lateral foramen; lvg, lateroventral groove; nc, neural canal; ns, neural spine; pa, parapophysis; pf, peduncular fossa; poz, postzygapophysis; pozdl, postzygodiapophyseal lamina; prz, prezygapophysis; przdl, prezygodiapophyseal lamina; przel, prezygoepipophyseal lamina; sprzl, spinoprezygapophyseal lamina.

The axial neural arch (MPCO.V 0067; Fig. 4a) lacks its cranial margin and is best preserved on its right side. Its ventral margin, which articulated to the centrum, is sigmoid in lateral view, concave cranially and convex caudally. The articular facet for the atlantal postzygapophysis is located at the craniodorsal corner of the neural arch and does not project laterally to form a pedicel-like postzygapophysis. It instead faces caudolaterally and, although its cranial third is missing, was likely ovoid in shape (craniocaudally oriented long axis in lateral view). Below that, the strongly ventrally bent diapophysis trends cranially. Although lacking its cranial edge, the diapophysis was likely subtriangular in (dorsolateral) profile, because its caudal margin is cranioventrally to caudodorsally oriented. This differs from the axial diapophysis of *Mas*. *knopfleri*^3^, which has a concave caudal margin and a cranial margin more caudally positioned relative to the prezygapophyseal facet. In fact, as far as it is possible to infer, the cranial margin of the axial diapophysis of *Ves*. *paranaensis* is flush with the cranial margin of the neural arch and not separated from its lateral surface by a deep notch as in *Mas*. *knopfleri*^3^. The surface of the infradiapophyseal fossa is perforated by two expansive pneumatic apertures; one more medially, cranially, and ventrally positioned, and another more laterally, caudally, and dorsally placed. These are separated by an oblique (craniodorsally to caudoventrally oriented) lamina and at least the latter aperture seems to invade the neural arch. Above and lateral to that opening, a raised portion of the neural arch extends as a ridge from the caudal margin of the diapophysis towards the caudoventral corner of the neural arch, probably representing an incipient version of the caudal centrodiapophyseal lamina and forming the caudodorsal boundary of the infradiapophyseal fossa. The neural arch is depressed between that incipient lamina and the postzygapophysis, but no pneumatic fossa is observable. A similar arrangement of laminae and fossae is seen below the postzygodiapophyseal lamina of *Mas*. *knopfleri*^3^. The ovoid postzygapophyseal facet of *Ves*. *paranaensis* is laterally expanded, caudoventrally oriented, and lateromedially concave for its entire craniocaudal extension. The dorsal surface of the postzygapophysis possesses a dorsoventrally flattened epipophysis, which expands well caudal to the facet, resembling the condition in *Li*. *inextricabilis* (Institute of Vertebrate Paleontology and Paleoanthropology, Beijing, China; IVPP V15923). In contrast, the axial epipophysis of *Mas*. *knopfleri* is much less projected caudally^3^. As a whole, the postzygapophysis expands caudodorsally at an angle of about 40° to the horizontal plane and the caudal margin of the neural arch below that is markedly concave in lateral view. The dorsal margin of the neural spine is sigmoid in lateral view, with a lateromedially expanded caudal portion. Its base extends from the cranial margin of the neural arch to surpass its caudal edge, forming a projection with a cranioventrally to caudodorsally oriented caudal margin, resembling the condition in *Mas*. *knopfleri*^3^. The caudal aperture of the neural canal is sub-rectangular, and slightly deeper than broad.

The best-preserved cranial postaxial neck vertebra, MPCO.V 0035 (Fig. 4b–g; see Supporting Information), has an hourglass-shaped centrum in ventral view, with cranial and caudal ends lateromedially expanded to about half the centrum length and a ca. 30% narrower central portion. The cranial end is somewhat broader lateromedially at its ventral part, due to the presence of the parapophyses, which expand cranioventrally from the cranioventral corner of the centrum. Yet, the articulation surfaces of the parapophyses are equally broad lateromedially. The ventral surface of the centrum is smooth, with no marked midline keel or groove, as is typical of Noasauridae^4^. A marked elliptical depression (“cvd” in Fig. 4d), with a lateromedial long axis, is present immediately caudal to the ventral margin of the cranial articulation facet, between the parapophyses. A pair of subtle grooves (“lvg” in Fig. 4d) extends craniolaterally from the midline of the caudal portion of the ventral surface of the centrum, entering its lateral surface and extending cranially in the direction of, but not reaching, a foramen located dorsal to the parapophyses. Between the caudal part of these grooves, a longitudinally elongated rugose area extends a short distance over the midline of the ventral surface of the centrum. The centrum is “parallelogram-shaped” in lateral view, although the cranial and caudal articular surfaces are not parallel, with the former facing much more ventrally than the latter faces dorsally, as also occurs in the cranial postaxial cervical vertebrae of *Mas*. *knopfleri*^3^ and an indeterminate noasaurid^69^ from the same unit (Lecho Formation) as *N*. *leali*. Furthermore, the cranial articular surface is dorsally displaced relative to the caudal, with the ventral margin of the centrum being subtly sigmoidal in lateral view: concave cranially and caudally concave. The caudal articulation is deeply excavated and has a sub-oval caudal outline, being slightly flattened dorsally and lateromedially broader than dorsoventrally deep. The cranial articular surface is nearly twice broader than deep and subtly concave at its centre, as is the case in *Mas*. *knopfleri*^3^. It has a pentagonal shape, with a dorsal base and ventrally converging lateroventral margins, the lateral portions of which are flanked by the parapophyses. Both lateral surfaces of the centrum bear a large ovoid pneumatic foramen dorsal to the parapohysis, resembling the condition in *Mas*. *knopfleri*^3^. This pneumatic foramen is absent, although a shallow blind fossa is present, in the cranial postaxial cervical vertebra of the indeterminate noasaurid from the Lecho Formation (Museo Argentino de Ciencias Naturales “Bernardino Rivadavia”, Buenos Aires, Argentina; MACN-Pv 662). In *Ves*. *paranaensis*, a broad ridge extends caudally from above the foramen to contact a larger, non-perforating depression (“ld” in Fig. 4c) in the caudal part of the centum, which bears a small nutrient foramen at its cranial margin. This is also seen in *Mas*. *knopfleri*^3^ and fits the position of the caudal pleurocoel of early theropods^70^. Another pneumatic foramen perforates the centrum at its mid-length, above the broad ridge and right below the neurocentral suture. The suture is partially closed at its cranial and, especially, caudal portions. Its remaining middle portion is composed of two nearly straight lines that converge dorsally to a summit below the diapophysis.

Each diapophysis expands lateroventrally from the lateral surface of the neural arch between the zygapophyses, tapering towards an area dorsal to the parapophysis. As the latter is located at the cranial part of the centrum, the diapophysis is subtriangular in dorsolateral profile, trending cranially at its lateral tip. Its dorsal surface is dorsolaterally arching and is slightly depressed at its centre. The position of the dia- and parapophysis suggests that the capitulum and tuberculum rib articulations were in close proximity. Each diapophysis is connected to the pre- and postzygapophyses by well-developed pre- and postzygodiapophyseal laminae. It is also connected to the craniodorsal corner of the centrum by the cranial centrodiapophyseal lamina, which corresponds to a mainly vertical pillar of bone. That centrodiapophyseal lamina is absent in the indeterminate noasaurid of the Lecho Formation (MACN-Pv 662). In addition, the caudal centrodiapophyseal lamina of MPCO.V 0035 extends craniodorsally from the caudodorsal corner of the centrum to intercept the middle part of the postzygodiapophyseal lamina. As seen in the best-preserved left side, the set of laminae radiating from the diapophysis defines the neural arch fossae. The infraprezygapophyseal fossa is set below the prezygodiapophyseal and cranial to the cranial centrodiapophyseal laminae. It faces mainly cranioventrally and is perforated by a pneumatic foramen that leads medially with a caudodorsal cant. The infradiapophyseal fossa is set between the centrodiapophyseal laminae and medial to the distal portion of the zygodiapophyseal laminae. It is perforated by three extensive pneumatic openings leading dorsally through the pedicel. The infrapostzygapophyseal fossa is set between the prezygodiapophyseal and the cranial centrodiapophyseal laminae and, unlike the fourth neck vertebra of *Mas*. *knopfleri*^3^, it is not perforated by any pneumatic structure. Both cranial and caudal openings of the neural canal are rounded and similar in size, i.e. about 2/3 the breath of the articular facets.

The prezygapophyses are incomplete, but expand craniolaterally from the neural arch at an angle of 20° in dorsal view. The postzygapophyses form slightly higher angles to the sagittal line in the same view. Their articular facets are transversely concave along their craniocaudal length. They face mainly ventrally, slightly caudally, and slightly laterally on their medial portions. The epipophysis is a constrained blunt ridge above each postzygapophysis and, unlike in *Mas*. *knopfleri*^3^, does not project caudal to the articular facet. The prezygoepipophyseal lamina corresponds to a subtle ridge on the dorsolateral edge of the neural arch. Most of the likely dorsoventrally short neural spine is missing, but its preserved base indicates that it extended over the caudal half of the neural arch. Cranial to that, the roof of the neural canal is exposed between the spinoprezygapophyseal laminae, forming a deep (deeper than lateromedially broad at its cranial end) fossa (“dcrf” in Fig. 4e), which continues caudally as the prespinal fossa on the cranial surface of the neural spine, as also occurs in the cranial postaxial cervical vertebrae of *Mas*. *knopfleri*^3^ and the indeterminate noasaurid of the Lecho Formation^69^. In dorsal view, the fossa takes the form of a craniocaudally elongated half-ellipse, that occupies nearly 1/3 of the craniocaudal length of the neural arch. In that same view, a subtriangular fossa (“dcf” in Fig. 4e) expands lateromedially towards its caudal end exposing the caudal part of the roof of the neural canal. The fossa is also subtriangular in caudal view, broader at the base, where two lateral furrows extend cranially into the neural arch, as also seen in *Mas*. *knopfleri*^3^ and the indeterminate noasaurid of the Lecho Formation^69^, with ventral diverticula extending into the pedicle. The flat surface between them is continuous to the postspinal fossa on the caudal surface of the neural spine.

MPCO.V 0048 (Fig. 4h–j) preserves only the centrum and the cranial half of the left neural arch. It matches the previously described vertebra in various aspects, but is 25% longer and its articulations are dorsoventrally deeper, although not significantly broader lateromedially. Other differences include cranial and caudal articulations set more perpendicularly relative to the long axis of the centrum in lateral view and subparallel to one another, resembling the condition in the mid-cervical vertebrae of *Mas*. *knopfleri*^3^ and *La*. *indicus*^9^. Yet, the cranial articulation is still slightly offset dorsally relative to the caudal. It is shallowly concave overall and has a hexagonal shape in cranial view, with a broader dorsal margin and ventrolateral margins that converge to a short ventral margin between the parapophyses. The cranial articular surface is approximately as tall as broad, contrasting with the transversely broader cranial articulations of the middle-cervical vertebrae of *Mas*. *knopfleri*^3^ and *La*. *indicus*^9^. The centrum is also more lateromedially compressed at its centre and its ventral surface is more concave in lateral view, bearing a subtle longitudinal ridge on its caudal portion flanked by a pair of equally subtle longitudinal fossae. Also, as in MPCO.V 0035, a lateromedially elongated depression is seen on the cranial margin of the ventral surface, slightly cranial to the parapophyses. MPCO.V 0048 is almost certainly from a more caudal part of the neck than MPCO.V 0035; respectively deriving from around the 5th–6th and 3rd–4th positions, when compared to *Mas*. *knopfleri*^3^. The lateral surface of the centrum of MPCO.V 0048 is excavated by a caudally tapering, subtriangular depression (“crd” in Fig. 4i) at its cranial third, dorsal to the parapophyses. It bears a more deeply excavated fossa on its cranial portion and a perforating foramen at its caudal tip. This fossa is at the same position as the cranial pneumatic foramina of the mid-cervical vertebrae of *Mas*. *knopfleri*^3^ and *La*. *indicus*^9^. As in *Mas*. *knopfleri*^2^, *N*. *leali* (Fundación “Miguel Lillo”, San Miguel de Tucumán, Argentina; PVL 4061), and *La*. *indicus*^9^, but unlike MPCO.V 0035, a shallow ovoid peduncular fossa (=laminopeduncular foramen^71^) is seen laterodorsal to the cranial opening of the neural canal. This also suggests a more caudal position for this vertebra, as the peduncular fossa gets better developed in middle neck vertebrae of *Mas*. *knopfleri*^2^.

MPCO.V 0034a-d represents a set of two disarticulated neural arches and centra (Fig. 4k–p; see Supporting Information), which match in size and possibly correspond to parts of the same two elements. One of the centra is elongate (MPCO.V 0034c; Fig. 4o), ventrally concave, with offset and non-parallel articulations, probably positioned around the 4–5th neck vertebrae. The other seems to represent a more caudal element (MPCO.V 0034d; Fig. 4p). It differs from the previous one in having more parallel articulations, less laterally projected parapophyses, a less concave ventral margin with a longitudinal midline ridge, and a maximal lateromedial compression displaced towards its cranial half. Both centra have two clear pneumatic openings on each of their lateral surfaces, with the one caudodorsal to the parapophyses being much larger. This resembles the condition in *La*. *indicus*^9^, and a caudal cervical vertebra of *Mas*. *knopfleri*^2^, in which the caudal aperture is, however, as large as the cranial. Indeed, the caudal of such foramina has been considered a ceratosaur synapomorphy^4^, but it is also present in non-averostran neotheropods (e.g. *Dilophosaurus wetherilli*: University of California Museum of Paleontology, Berkeley, USA; UCMP 37302). As with MPCO.V 0048, MPCO.V 0034c is three times craniocaudally longer than its maximum dorsoventral depth, a character considered synapomorphic for the mid-cervical vertebrae of Noasauridae^4^.

Compared to MPCO.V 0035, the best-preserved neural arch of MPCO.V 0034 (MPCO.V 0034a; Fig. 4k–m) has a caudal centrodiapophyseal lamina reaching its cranial summit closer to the diapophysis, which is less ventrally pendant. As such, it resembles the mid-cervical elements of *Mas*. *knopfleri*^2^, as well as the only known cervical vertebra of *N*. *leali*^5^. It was detached from its centrum along the neurocentral suture, which is composed of two nearly straight lines that converge dorsally below the caudal margin of the diapophysis. The zygodiapophyseal laminae are as developed as those of MPCO.V 0035, but the cranial centrodiapophyseal lamina is less pillar-like and more horizontally oriented. Both infrazygapophyseal fossae are well developed and perforated by a large pneumatic opening leading to the centre of the neural arch, with a small subsidiary foramen in the case of the right infrapostzygapophyseal fossa. Likewise, two large openings perforate the dorsal surface of the infradiapophyseal fossa. There is no recognisable peduncular fossa, contrasting with those seen in *Mas*. *knopfleri*^2^, *N*. *leali* (PVL 4061), and *La*. *indicus*^2,9^. In dorsal view, the neural arch of MPCO.V 0034a is elongated (nearly twice longer that maximally broad), waisted at its middle part, and bears equally laterally projected zygapophyses. As such, it more resembles the mid-neck vertebrae of *Mas*. *knopfleri*^2^ than those described for both *N*. *leali*^5^ and *La*. *indicus*^2,9^. Yet, given the non-elevated prezygapophyses, it probably still comes from the cranial half of the neck, around the 4–5th positions, although more caudally positioned than MPCO.V 0035. The dorsal surface of its diapophysis is markedly depressed and medially bounded by a ridge that extends caudally from the caudolateral corner of the prezygapophyses. This ridge forms the angled dorsolateral margin of the cranial half of the neural arch, as is typical of abelisauroids^4^. The ridge faints caudal to the diapophysis, merging towards the postzygodiapophyseal lamina. Although this ridge is surely homologous to the prezygoepipophyseal lamina, it does not reach the epipophysis, as it does in the mid-cervical vertebrae of *Mas*. *knopfleri*^3^ and the available cervical vertebrae of *N*. *leali*^5^ and *La*. *indicus*^2,9^, leaning instead towards the lateral margin of the postzygapophiseal facet. In dorsal view, the prezygapophyses expand craniolaterally at an angle of ca. 20° to the sagittal line, whereas the postzygapophyses form a slightly smaller angle. The ovoid prezygapophyseal facets face mostly dorsally, with minor cranial and medial windings, resembling the condition in *N*. *leali*^5^ and the 4th cervical vertebra of *Mas*. *knopfleri*^3^. In contrast, this differs from the larger and more strongly angled prezygapophyses of *La*. *indicus*^2,9^. The postzygapophyseal facets nearly mirror the prezygapophyseal arrangement, facing mostly ventrally, but also slightly caudally and laterally. Each postzygapophysis is covered by a plate-like epipophysis that extends caudal to the articular facet for about half of its craniocaudal length, contrasting with the proportionally longer and more dorsally projected epipophyses of *N*. *leali* (PVL 4061). The well-developed and tapering cranial projection observed on the prezygoepipophyseal lamina of *N*. *leali*^6^ is not present in any of the available cervical vertebrae of *Ves*. *paranaensis*, *Mas*. *knopfleri*^3^, and *La*. *indicus*^2,9^. The neural spine expands only slightly dorsally, occupying only about 1/5 the craniocaudal length of the neural arch, in a position that probably corresponds to the cranial part of the centrum, as is typical of noasaurines^2,4^. It is cranially bordered by the fossa located between the spinoprezygapophyseal laminae, but does not reach the corresponding caudal fossa, which is broader and more “V-shaped” in dorsal view. The second neural arch of MPCO.V 0034 (MPCO.V 0034b; Fig. 4n), is more fragmentary and lacks its cranial half. It matches the above described arch in most details, although the postzygapophysis expands caudally with a more marked dorsal inflection, around 30° to the horizontal plane, suggesting a more caudal position in the neck. The epipophyses are narrower lateromedially, with a more oblique long axis when viewed caudally. They extend cranially as a dorsal ridge, not observed in MPCO.V 0034a, but it is unclear if it connected to the prezygapophysis. As typical of noasaurids^4^, the epipophyses are not dorsally expanded, but do not approach the caudally sharply pointed morphology seen in the mid-cervical vertebrae of *Mas*. *knopfleri*^2^ and *N*. *leali*^6^.

**Figure 5:**
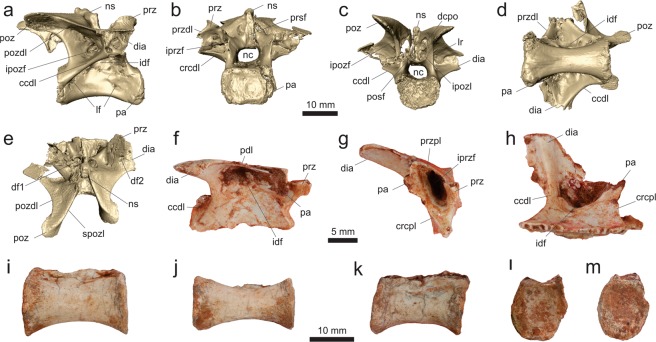
Caudal neck and trunk vertebrae of *Vespersaurus paranaensis* gen. et sp. nov. (**a–e**) Caudal neck vertebra (MPCO.V 0017) in right lateral (**a**), cranial (**b**), caudal (**c**), ventral (**d**), and dorsal (**e**) views. (**f–h**) Trunk neural arch (MPCO.V 0066) in lateral (**f**), cranial (**g**), and ventral (**h**) views. (**I**,**j**) trunk centrum 0065d10b (holotype) in lateral (**i**) and ventral (**j**) views; (**k**–**m**) trunk centrum 0065d10a (holotype) in lateral (**k**), cranial (**l**), and caudal (**m**) views. Anatomical abbreviations, as in Fig. 4, plus: crcdl, cranial centrodiapophyseal lamina; crcpl, cranial centroparapophyseal lamina; dcpo, dorsocaudal pneumatic opening; df1&2, dorsal foramina 1 and 2; ipozl, intrapostzygapophsyeal lamina; lr, lateral ridge; pdl, paradiapophyseal lamina; posf, postspinal fossa; prsf, prespinal fossa; przpl, prezygoparapophyseal; spozl, spinopostzygapophyseal lamina.

MPCO.V 0017 corresponds to a caudal neck vertebra (Fig. 5a–e; see Supporting Information), around 8–9th positions if compared to the presacral series of *Mas*. *knopfleri*^2,3^. The centrum is elongate, nearly 2.5 times longer than deep. In lateral view, the ventral surface is concave, and the articular facets are parallel to one another and positioned at the same dorsoventral plane. There is no marked groove or keel on the ventral surface of the centrum, although a subtle ridge is present within its cranial half, being broader cranially and flanked by an equally subtle pair of elongate depressions. Both articulations are shallowly concave and about 1.5 broader than deep. Their cranial and caudal outlines are, respectively, subrectangular and crescent-shaped (concave dorsally and convex along the rest of its margins). The parapophyses are located at the cranioventral corners of the centrum, which has depressed lateral surfaces. Together with the flattened ventral surface, this results in a subrectangular vertebral body in cross section, with angled lateroventral edges. Better evidenced on the left side, a pair of pneumatic apertures pierce each side of the centrum at its cranio- and caudodorsal corners, resembling the condition of the caudal cervical vertebrae of *Mas*. *knopfleri*^2^. The neurocentral junction is still recognisable as a pair of nearly straight suture lines that converge dorsally towards the middle of the vertebra.

The neural arch is marked by the presence of robust diapophyses, both of which have broken lateral ends. The broken surfaces of the diapophyses expose the highly pneumatic internal structure of the neural arch, which is composed of large chambers separated by trabeculae. As preserved, each expands laterally, with no meaningful caudal or ventral inflections, but with a minor cranial inflection, and its caudal margin is positioned at the mid-length of the centrum. Each diapophysis is surrounded by the typical saurischian laminae and fossae complex. The well-developed postzygodiapophyseal and caudal centrodiapophyseal laminae surround a deep infrapostzygapophyseal fossa. This opens caudolaterally and is perforated by a main/central pneumatic aperture that leads medially with a craniodorsal inflection towards the neural arch. Inside the fossa, smaller blind pockets are positioned both dorsal and ventral to the aperture. As best seen in the left side, the shallower infradiapophyseal fossa is perforated by cranial, dorsal, and caudal foramina, the former of which is significantly larger, leading craniomedially into the neural arch. A similar pattern, but not identical in terms of size and position of the apertures, is seen on the right side. Unlike its caudal counterpart, which reaches the caudodorsal corner of the centrum, the cranial end of the cranial centrodiapophyseal lamina is more dorsally positioned. Indeed, it does not reach the craniodorsal corner of the centrum, but intercepts the cranial margin of the neural arch somewhat dorsal to that. A very similar pattern was described for a caudal cervical vertebra of *Mas*. *knopfleri*^2^. Consistent with the minor cranial projection of the prezygapophyses, the prezygodiapophyseal lamina is relatively short. As a result, the infraprezygapophyseal fossa, formed between the two previously mentioned laminae is dorsally displaced and relatively small. It is medially bordered by the dorsal half of the pedicle (=centroprezygapophyseal lamina^2^) and, although not well preserved in either side, it appears to have hosted a deep pneumatic furrow penetrating caudomedially into the neural arch, as described for *Mas*. *knopfleri*^2^. There is no peduncular fossa, as is the case in the caudal cervical vertebrae of *Mas*. *knopfleri*^2^.

Both cranial and caudal apertures of the neural canal are ovoid, ca. 1.5 times broader than deep, and much smaller than the respective centrum articulation. The neural arch is rather craniocaudally extensive at its base, its ventral articulation spreading over about 80% the craniocaudal length of the centrum, narrowing along that axis as it expands dorsally towards the zygapophyses. Although positioned nearly at the same dorsoventral level, the zygapophyses are structured very differently from one another, as is the dorsal surface of the neural arch between them and the neural spine. The prezygapophyses do not expand cranial to the cranial margin of the centrum, as occurs in *Mas*. *knopfleri*^2^. In fact, their articular facet is not much more than a cranial expansion of the margin of the bone that extends craniolaterally (forming an angle of 45° to the sagittal line in dorsal view) between the cranial ends of the neural spine and the diapophysis. The prezygapophyseal facet is ovoid, with a craniocaudal long axis. It is oriented dorsomedially (forming an angle of about 45° to the vertical/horizontal axis in cranial view), but also slightly caudally. The dorsal surface of the neural arch caudomedial to the prezygapophyses is strongly depressed. It then rises both medially and caudally to form the nearly vertical lateral surfaces of the neural spine. The area flanking the neural spine is excavated by a deep dorsoventrally elongated pneumatic pocket (“df1” in Fig. 5e), the ventral portion of which perforates towards the roof of the neural canal, as seen in *Mas*. *knopfleri*^2^. Another pneumatic aperture (“df2” in Fig. 5e), is positioned caudolateral to the prezygapophyseal facet, perforating caudomedioventrally the dorsal surface of the diapophysis. In cranial view, bordered laterally by the above-mentioned pocket and depressed surface, it is possible to see that, in contrast to more cranial neck vertebrae, the neural spine is dorsoventrally extensive, corresponding to half the depth of the neural arch, resembling the condition in *Mas*. *knopfleri*^2^. Its cranial surface is excavated by a shallow and (following the shape of the spine) dorsoventrally elongated prespinal fossa. This is likely related to the attachment of the interspinous ligament, which may also account for the lateromedial compression of the craniodorsal portion of the spine. In lateral view, the neural spine is caudodorsally oriented and its cranial margin forms an angle of about 75° to the horizontal. This is also the case of its caudal margin, which is excavated by an equally elongated, but slightly deeper postspinal fossa, which is also possibly related to the attachment of the interspinous ligament. The distal end of the neural spine has two transverse clefts that define a central prominence. The presence of a single transverse cleft is present in the caudal cervical and cranial dorsal vertebrae of *Mas*. *knopfleri*^2^ and *Majungasaurus crenatissimus*^71^. As occurs in the above-mentioned species, the distal end of the neural spine of *Ves*. *paranaensis* is lateromedially constricted in dorsal view at the level of the transverse clefts. The base of the neural spine occupies only the cranial half of the roof of the neural canal, with its dorsal margin located caudal to the mid-length of the centrum. In dorsal view, the roof of the neural canal has mirroring cranial and caudal margins, so that the latter is caudolaterally oriented at an angle of about 45° to the sagittal line. As a consequence, only about 50% of the neural canal is roofed at the midline. All those medial elements of the caudal portion of the neural arch are laterally covered by the strongly caudodorsally expanded postzygapophyses and associated laminae, which extend well beyond the level of the caudal margin of the centrum. As such, the caudal margin of the neural arch, including the part formed by the postzygapophyseal extension above the roof of the neural canal, is markedly concave in lateral view, resembling the condition in *Mas*. *knopfleri*^2^. The oval postzygapophyseal articular facets have a craniocaudal long axis and face caudolaterally, forming a 45° angle to the horizontal/vertical planes. The dorsal surface of each postzygapophysis is covered by a strong ridge, which is probably homologous to the spinopostzygapophyseal lamina^72^. As such, there is no prezygoepipophyseal lamina. The ridge extends craniomedially, reaching the caudal margin of the neural spine at its dorsal tip. The surface lateral to that is elevated relative to more cranial areas. Ventromedial to the ridge, a vertical wall forms the medial surface of the postzygapophysis above the roof of the neural canal. This is delimited cranially by the caudal margin of the neural spine and ventrally by the roof of the neural canal caudal to the spine. This wall is, however, nearly entirely excavated by a pneumatic opening (“dcpo” in Fig. 5c) leading cranially to several chambers that fill the space between the postzygodiapophyseal lamina and the concealed lateral surface of the neural spine, also penetrating the pedicle (ventrally) and the diapophysis (laterally). In caudal view, two ridges extend ventrally from the postzygapophyseal facet. The more lateral (“lr” in Fig. 5c) reaches the dorsolateral corner of the centrum, its ventral half representing the caudal margin of the lateral wall (pedicle) of the neural canal. The medial ridge corresponds to the intrapostzygapophsyeal lamina. It extends halfway down and turns medially to continue as the caudal margin the roof of the neural canal and meet its counterpart. A subtle depression is formed between the two ridges, which is not pneumatically perforated as in *Mas*. *knopfleri*^2^.

#### Trunk

The trunk series of *Ves*. *paranaensis* is represented only by one isolated partial neural arch (MPCO.V 0066; Fig. 5f–h) and some isolated centra (e.g. MPCO.V 0010, 0040, 0062a-b), including three preserved in the holotypic block (MPCO.V 0065d10a-c; Fig. 5i–m). The centra are all very simple elements, ventrally concave in lateral view, lateromedially constricted at their mid-length, and lacking midline ridges or a groove on their ventral surface. They are usually more than 1.5 times longer than deep, as is typical of Noasauridae^4^, with the cranial and caudal articulations both concave and somewhat deeper than broad. These traits compare well with those of the middle-caudal trunk vertebra of *Mas*. *knopfleri*^3^, a referral also bolstered by the absence of parapophyseal facets in the centra, which comparatively indicates positions caudal to the 4th trunk element. A longitudinally elongated depression is present on their lateral surface, cranially deeper in some elements, but the pneumatic apertures seen in the neck vertebrae, and in some cranial trunk vertebra of *Mas*. *knopfleri*^3^, are lacking. The alternative referral of these centra to the proximal part of the tail, with *Mas*. *knopfleri*^3^ bearing very similar vertebrae in this region to the caudal part of the trunk, is tentatively dismissed given the absence of evident haemal arch facets and the somewhat more gracile morphology of the lateral and ventral margins of the articulation facets^4^. It is worth noting, however, that the lateromedial constriction of some centra better fits the expected morphology of tail vertebrae.

The partial neural arch MPCO.V 0066 compares better to those of the middle part of the trunk series of *Mas*. *knopfleri*^3^. Only the central part of the right neural arch is preserved, lacking the roof of the neural canal, postzygapophysis, and neural spine. The diapophysis is dorsolaterally and slightly caudally directed, and positioned on the caudal half of the neural arch. In ventral/dorsal views, the diapophysis becomes craniocaudally narrower as it expands laterally to end in a straight margin for the capitular articulation. Although incomplete, the parapophysis is positioned well above the neurocentral junction on the cranial margin of the neural arch, somewhat below the level of the diapophysis. A sharp cranial centroparapophyseal lamina extends ventrally from the parapophysis to reach the cranioventral corner of the neural arch. The caudal centroparapophyseal lamina described for *Mas*. *knopfleri*^3^ cannot be observed, although a ridge extends caudoventrally from the parapophysis. The cranial centroparapophyseal lamina laterally hides a very deep infraprezygapophyseal fossa, which is clearly visible in cranial view, lateral to the prezygapophysis. The prezygapophysis extends further cranially than the ventral margin of the neural arch and is connected to the parapophysis by the prezygoparapophyseal lamina, which covers the infraprezygapophyseal fossa. The articular face is small, ovoid, and faces dorsally. The parapophysis is connected to the diapophysis by the paradiapophyseal lamina, which roofs the infradiapophyseal fossa. The caudal margin of this fossa is formed by a strong caudal centrodiapophyseal lamina, which buttresses the diapophysis as a vertical pillar, its ventral portion bowing caudally towards the caudoventral corner of the neural arch. A poorly preserved infrapostzygapophyseal fossa excavates the caudal surface of the caudal centrodiapophyseal lamina, dorsolateral to the preserved part of the neural arch pedicle. It is not clear if the fossa possessed pneumatic apertures.

#### Sacrum

**Figure 6:**
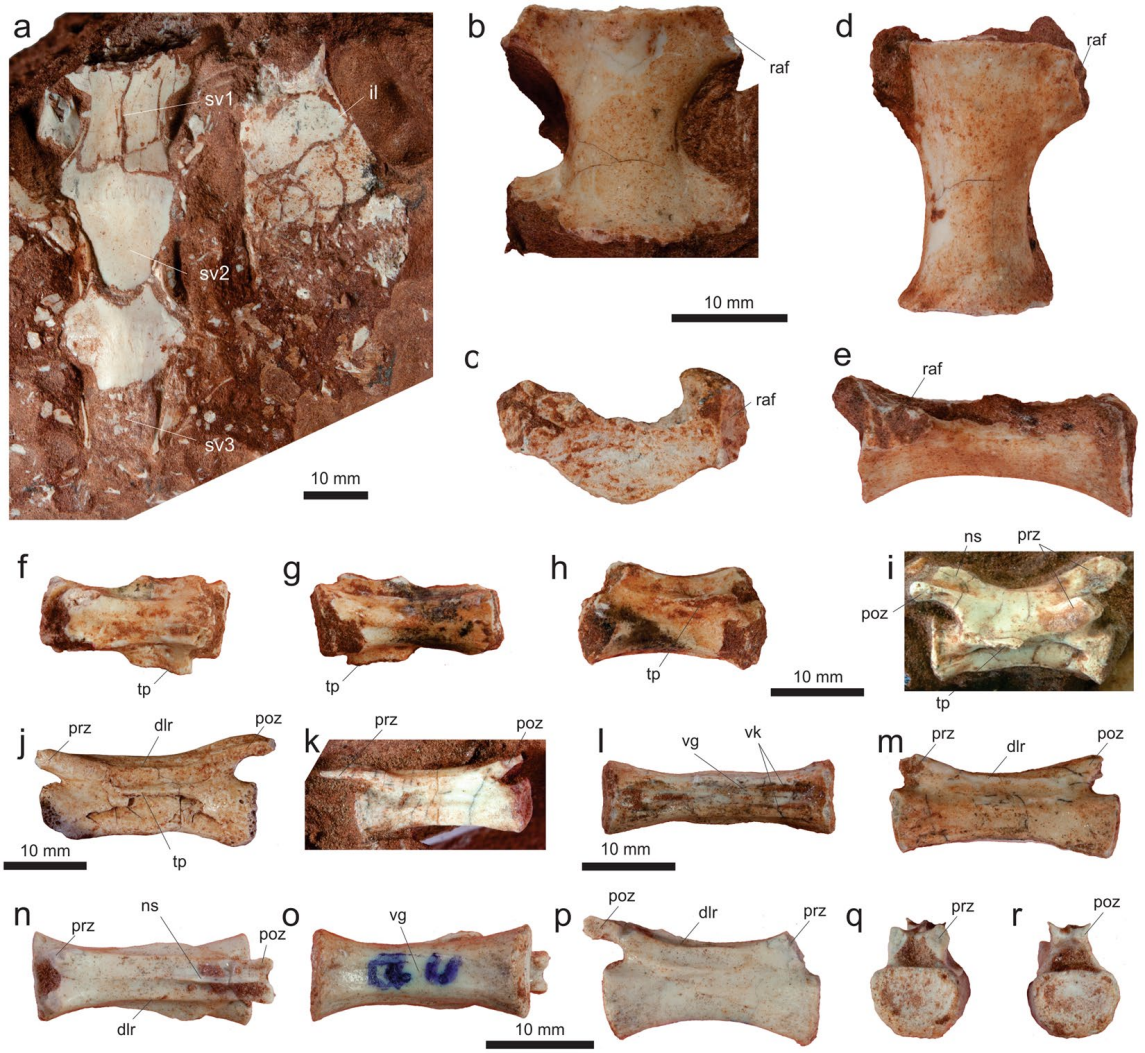
Sacral and tail vertebrae of *Vespersaurus paranaensis* gen. et sp. nov. (**a**) Sacral vertebrae (MPCO.V 0064a) in ventral view. (**b,c**) Sacral vertebra (MPCO.V 0065d7) in ventral (**b**) and cranial (**c**) views. (**d,e**) Sacral vertebra (MPCO.V 0065d6, holotype) in ventral (**d**) and left lateral (**e**) views. (**f**–**h**) Tail vertebra (MPCO.V 0065d9, holotype) in dorsal (**f**), ventral (**g**) and left lateral (**h**) views. (**i**) Tail vertebra (MPCO.V 0065d5, holotype) in right lateral view. (**j**) Tail vertebra (MPCO.V 0052) in left lateral view. (**k**) Tail vertebra (MPCO.V 0020) in left lateral view. (**l**–**m**) Tail vertebra (MPCO.V 0024) in ventral (**l**) and dorsal (**m**) views. (**n**–**r**) Tail vertebra (MPCO.V 0025) in dorsal (**n**), ventral (**o**), right lateral (**p**), cranial (**q**), and caudal (**r**) views. Anatomical abbreviations, as in Fig. 4, plus: dlr, dorsolateral ridge; il, ilium; raf, rib articulation facet; sv1-3, sacral vertebrae 1–; tp, transverse process; vg, ventral groove; vr, ventral ridge.

Sacral vertebrae are represented by three isolated centra (MPCO.V 0065d6-8) from the holotypic block, as well as by an articulated series of three centra (MPCO.V 0064a; Fig. 6a) exposed in ventral view within the same block as the partial hand. The articulated sacral centra are completely co-ossified to one another, as indicated by the longitudinal striations on the lateromedially expanded portions of the series. This indicates that the cranialmost vertebra of the series (“sv1” in Fig. 6a) lacks its cranial margin and that only the cranial portion of the caudalmost element (“sv3” in Fig. 6a) was preserved. As seen in *Mas*. *knopfleri*^2^ and other neotheropods, sacral ribs are shared by adjacent centra in MPCO.V 0064a. The craniocaudal length of the most completely preserved centrum is more than twice its transverse width at mid-length. All elements have a concave ventral margin in lateral view and a transversely rounded, smooth ventral surface, with no midline groove or ridge. Based on the relative position of the portion of ilium preserved alongside the vertebrae and in comparison to *Mas*. *knopfleri*^2^, these most probably correspond to sacral vertebrae 1−3. Also, with respect to the more complete holotypic ilium, there would be space for three extra sacral elements medial to the iliac postacetabular ala. Accordingly, a six-vertebrae sacrum, as seen in *Mas*. *knopfleri*^2^ and generally typical of ceratosaurs^4^, seems likely for *Ves*. *paranaensis*.

The three isolated elements of the holotypic sacral vertebrae (Fig. 6b–e) are preserved disarticulated among the ilium, ischium, and foot. This indicates that the centra were not co-ossified at the time of death, which is consistent with their smaller size (about 30%) compared to those of MPCO.V 0064a. Likewise, as only the centra are preserved, the neurocentral junction was probably also not fully closed. The centra are all about twice longer than lateromedially broad at their mid-length, but expand laterally at both the cranial and caudal margins. The cranial margin is typically the broader of the two, being almost the length of the centrum in two of the elements, and reaching that length in one of them. These lateral expansions are related to the rib articulations, which are always shared between consecutive centra. As such, their craniolateral corners are flattened, forming craniolaterally facing articular facets for the ribs. The centra are dorsoventrally low, with their articulation facets lower than half of their craniocaudal lengths. Accordingly, the facets are also about twice broader (lateromedially) than deep. Most of these are flat and crescent-shaped in outline, concave dorsally and convex along the other margins. The lateral surface of the centra are depressed, but lack pneumatic apertures. Their ventral surfaces are concave in lateral view and lack a midline ridge or groove. Similar to *Mas*. *knopfleri*^4^, the preserved sacral vertebrate indicate that *Ves*. *paranaensis* did not possess the transversely compressed, rod-like middle sacral centra see in other ceratosaurs.

#### Tail

Caudal vertebrae in the *Ves*. *paranaensis* assemblage include three disarticulated elements from the holotype (MPCO.V 0065d4-5, 0065d9), as well as several isolated or clustered bones (MPCO.V 0020, 0024, 0025, 0026, 0027, 0029, 0052, 0061). Their centra are all amphicoelous and more than twice (often three times) longer than dorsoventrally deep. There is no evidence of vertebral pneumatization and the neural arches lack laminae. Compared to *Mas*. *knopfleri*^2,3^, this indicates that the preserved vertebrae belong to the middle and distal portions of the tail. There is no sign of the neurocentral junction in any of the tail vertebrae. The best-preserved holotypic element (MPCO.V 0065d5; Fig. 6i) is very similar to the figured mid-caudal vertebra of *Mas*. *knopfleri*^2^. The centrum is nearly three times longer than deep at its proximal and distal articulations. Its ventral margin is concave in lateral view and the distal facet for the chevron articulation is more marked than the proximal. The neural arch corresponds to more than half the dorsoventral depth of the vertebra at its mid-length. The dorsoventrally flattened transverse process expands laterally with a slight ventral cant. Although distally broken, its preserved base shows that the process was slightly caudally oriented and placed above the caudal half of the centrum. The prezygapophysis is proximodorsally oriented at an angle of about 40° to the horizontal axis and surpasses the proximal margin of the centrum. Compared to the prezygapophyses, the postzygapophyses are less dorsally oriented, but are dorsally arched. Their articular facets face medially and slightly dorsally. The ridge that connects the dorsal margins of the zygapophyses is more marked towards the postzygapophysis, nearly disappearing along the middle of the neural arch. No vestige of a distinct epipophysis is present. The neural spine extends over nearly the entire midline of the neural arch, corresponding to a faint ridge along its proximal part and expanding dorsally within its distal third. It does not display the marked dorsal rise seen in the corresponding vertebrae of *Maj*. *crenatissimus*^71^, resembling more the condition in *Mas*. *knopfleri*^2^. The two other tail vertebrae of the holotype, especially the more fragmentary MPCO.V 0065d9 (Fig. 6f–h), are very similar to the aforementioned. In the latter vertebra, it is possible to identify a subtle double-keel on the ventral surface of the centrum (area that is covered with matrix in the previously described vertebra). MPCO.V 0065d4, in contrast, has a less elongated centrum (two times longer than deep) and a more proximally extensive transverse process, likely representing a more proximal element. Its postzygapophyses are less dorsally arched compared to MPCO.V 0065d5. Its facet is ovoid and lateroventrally facing. The centrum and the aperture of the neural canal are circular in proximal view, but the latter is twice smaller.

All the other caudal vertebrae referred to *Ves*. *paranaensis* (Fig. 6j–r) come from more distal portions of the tail compared to those of the holotype. They are generally more elongate and their centra have a strong midline groove in the ventral surface, laterally flanked by a double ventral keel, as in *Mas*. *knopfleri*^2^. The neural spine and transverse processes take the form of distally displaced inconspicuous ridges, more marked in more proximal elements. The chevron articulation facets are subtle and some centra have striations surrounding the proximo- and distoventral corners. The more proximal of these elements (Fig. 6j,n–r) have less elongate centra (about three times longer than deep), with more strongly concave ventral margins in lateral view. They are also less lateromedially compressed, with proximal/distal articulations typically broader lateromedially than dorsoventrally deep. The neural arches also lack a mid-length constriction and bear marked ridges (“dlr” in Fig. 6n,p) extending along their dorsolateral edges between the zygapophyses. The better preserved prezygapophyses extend proximal to the proximal margin of the centrum for a distance corresponding to 15% the centrum length, whereas the articular facet of the only complete postzygapophysis forms a 30° angle to the vertical plane. More distal tail centra (Figs k–m) are more elongated, nearly four times longer than deep, as well as being more lateromedially compressed. This is seen in the proximal/distal articulations, which are dorsoventrally deeper than lateromedially broad in some elements. The neural arch is also lateromedially constricted between the zygapophyses. The only completely preserved prezygapophysis extends proximal to the proximal margin of the centrum for a distance corresponding to 20% the centrum length.

#### Pectoral girdle

**Figure 7:**
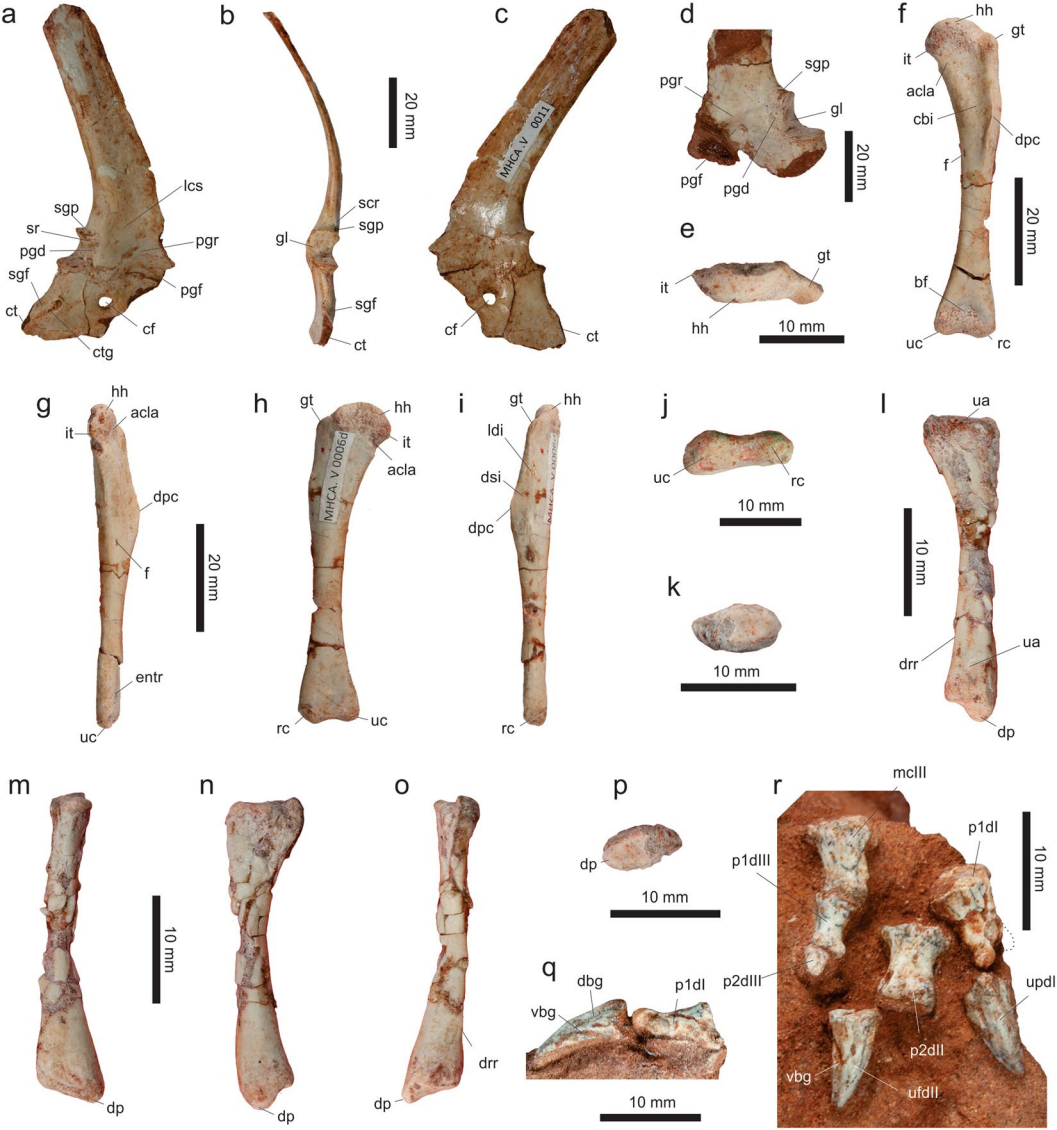
Pectoral girdle and limb elements of *Vespersaurus paranaensis* gen. et sp. nov. (**a–c**) Right sacapulocoracoid (MPCO.V 0011) in lateral (**a**), caudal (**b**), and medial (**c**) views. (**d**) Left sacapulocoracoid (MPCO.V 0006 f) in lateral view. (**e–j**) Left humerus (MPCO.V 0006d) in proximal (**e**), cranial (**f**), medial (**g**), caudal (**h**), lateral (**i**), and distal (**j**) views. (**k**–**p**) Left radius (MPCO.V 0006j) in proximal (**k**), medial (**l**), caudal (**m**), lateral (**n**), cranial (**o**), and distal (**p**) views. (**q**) Right manual digit I (MPCO.V 0006j) in medial view. (**r**) Right manus (MPCO.V 0006j) in dorsal view. Anatomical abbreviations: acla, acrocoracohumeral ligament attachment; bf, brachial fossa; cbi, M. coracobrachialis insertion; ct, coracoid foramen; ct, coracoid tuber; ctg, groove on coracoid tuber; dbg, dorsal blood groove; dp, descending process; dpc, deltopectoral ridge; drr, distal radial ridge; dsi, M. deltoideous scapularis insertion; entr, entepicondylar ridge; f, foramen; gl, glenoid; gt, greater tubercle; hh, humeral head; it, internal tuberosity; lcs, lateral concave surface; ldi, M. latissimus dorsi insertion; mcIII, metacarpal III; p1-2dI-III, phalanges 1–2 of manual digits I-III; pgd, preglenoid depression; pgf, preglenoid fossa; pgr, preglenoid ridge; rc, radial condyle; scr, scapular ridge; sgd, subglenoid fossa; sgp, supraglenoid pit; sr, stout ridge; ua, ulnar articulation; uc, ulnar condyle; updI-II, ungual phalanges of manual digits I-II; vbg, ventral blood groove.

A pair of right and left scapulocoracoids (respectively MPCO.V 0011, Fig. 7a–c, and MPCO.V 0013) of approximately the same size are preserved. However, given some minor anatomical differences, these probably come from different individuals. Both lack the distal portion of the scapular blade, the cranial margin of the acromial area, and the cranial half of the coracoid. Additionally, the glenoid area of a slightly smaller left scapulocoracoid (MPCO.V 0006f, Fig. 7d), probably represents a younger individual. The latter was found near a left humerus (MPCO.V 0006d), with which it probably articulated, given that the humeral head and the glenoid have matching size/shape. The larger left scapula and coracoid show a complete fusion between the two bones, whereas traces of the suture line are still observable in the right pectoral bones. Both scapula and coracoid bow laterally in cranial/caudal views, as does the overall scapulocoracoid, fitting the outer curvature of the ribcage. Yet, a slight medial deflection occurs at near the junction of the two bones.

MPCO.V 0013 bears preglenoid ridge and fossa^73^ that are much better developed than in the other two specimens. When the former is aligned to the horizontal, the scapular blade expands caudodistally from the scapular body at an angle of ca. 45°, as also is the case in MPCO.V 0011. In both specimens, the cranial margin of the scapular blade is incomplete in the ventral fourth of its preserved length. Distal to that, the cranial and caudal margins of the blade are almost parallel, so that its craniocaudal breadth right above its incomplete cranioventral margin is only about 15% higher than at its most distally preserved portion. Accordingly, the craniocaudal narrowing of the blade seen in *Li*. *inextricabilis*^57^ and *Mas*. *knopfleri*^3^, as well as the caudal angle formed at the distal portion of the blade in those taxa, is not present in *Ves*. *paranaensis*. However, as seen in *Mas*. *knopfleri*^3^, a subtly concave surface (“lcs” in Fig. 7a) is present above the preglenoid ridge, displaced somewhat cranially along the lateral surface of the blade and directly caudal to its incomplete cranial margin. Caudal to that, the scapular blade thickens lateromedially. In MPCO.V 0013, the caudal margin of the blade above the glenoid is transversely rounded. In contrast, the other scapulae possess a ridge (“scr” in Fig. 7b) extending dorsomedially from the dorsolateral corner of the glenoid, so that the caudal margin of the blade is sharper at that point. Lateral to the ridge and distal to the glenoid, a supraglenoid pit is present in all specimens, probably representing the attachment of the scapular head of M. triceps^74^. The supraglenoid pit is aligned directly caudal to the preglenoid ridge. In MPCO.V 0011, a stout ridge (“sr” in Fig. 7a) extends cranially from the dorsolateral corner of the glenoid, along the lateral surface of the scapula. It forms a rugose ventrolateral edge for the supraglenoid pit, also delimiting the distal margin of a dorsoventrally elongated depression (“pgd” in Fig. 7a) positioned cranial to the lateral margin of the glenoid, a subtler version of which is seen in MPCO.V 0013. The scapular glenoid of all specimens has everted, lip-like borders, which are particularly marked in the distal and lateral margins, especially so in the case the two larger specimens. The strong distal lip produces a caudoventrally facing articular surface, which is excavated by a fossa, especially more ventrally. Its caudal outline is similar among the specimens (dorsoventrally deeper than lateromedially broad in MPCO.V 0013 and 0006F, and as deep as broad in MPCO.V 00011), being slightly concave at the coracoid suture, convex both distally and medially, and sigmoid laterally; i.e. convex distally and concave ventrally. The latter convexity is, however, more marked/angled in MPCO.V 0011, as a result of bearing the stout ridge mentioned above. The ventral margin of the scapular glenoid is also more markedly concave in that specimen, with the medial portion of the scapula-coracoid suture expanding caudally, to almost conceal the coracoid contribution to the glenoid in medial view. In fact, as in *Mas*. *knopfleri*^3^, the ventral margin of the scapular glenoid in all specimens of *Ves*. *paranaensis* is more caudally expanded at its medial portion, resulting in a somewhat laterally facing articulation.

The lateral margins of the glenoid formed by the scapula and coracoid meet at an angle slightly higher than 90° in lateral view. Traceable on the lateral surface of MPCO.V 0011 only, the scapula-coracoid suture extends cranially from that junction as a nearly straight line, forming an angle somewhat over 90° to the scapular part of the glenoid. The suture is less clear on the medial side, but appears to mirror its lateral expression more cranially, whereas the caudal part arches ventrally to intersect the glenoid at its ventral tip, as apparently also seen in *Ceratosaurus dentisulcatus*^73^. A ventrally arched striated area is also seen in the medial surface of MPCO.V 0013. In general, the medial outline of the glenoid of *Ves*. *paranaensis* is more rounded than the lateral, which angles at the junction of the bones.

The incomplete cranial and ventral margins of the best-preserved coracoid preclude an estimation of its general shape. Based on the available evidence, however, it seems to fit the “high and short” condition considered synapomorphic of Noasauridae^4^. The coracoidal part of the glenoid has an ovoid outline and bears lip-like caudal and lateral margins. The articular surface is mostly flat, occupying the lateroventral third of the entire glenoid. It faces almost strictly dorsally, with subtle caudal and lateral orientations. It is clear from MPCO.V 0011 that the coracoid foramen is set well below the scapula-coracoid suture, extending caudodorsally as it perforates inwards. It is larger and further from the scapula compared to those of *Mas*. *knopfleri*^3^ and *Li*. *inextricabilis*^57^. The equivalent area in MPCO.V 0013 is fractured, and thus it is not possible to confirm if it housed such a perforation. The caudal margin of the coracoid extends ventrally as a nearly straight line, forming an angle slightly above 90° with the scapular suture (as seen in lateral view of MPCO.V 0011). This differs from the U-shaped notch considered synapomorphic of Noasauridae^4^, as present in *Mas*. *knopfleri*^3^, *Li*. *inextricabilis*^57^, and *E*. *bambergi*^4^. Indeed, the coracoid tuber (=posteroventral process) is present as a subtle caudal inflection within the ventral half of the caudal margin of the coracoid, where the bone tapers (lateromedially) distally, and does not project as caudally as those of other members of the group (e.g. *Mas*. *knopfleri*^3^; *E*. *bambergi*^4^; *Li*. *inextricabilis*^57^). The coracoid displays its maximal non-glenoidal thickness dorsal to the coracoid tuber through to the ventral margin of the glenoid, whereas the remainder of the bone is more laminar, being medially depressed and laterally convex. A peculiar, drop-shaped groove (“ctg” in Fig. 7a) extends along the lateral surface of the coracoid. Paralleling the caudal margin of the coracoid tuber, but cranially displaced from it, the groove has a more expanded, rounded craniodorsal portion, from which it tapers caudoventrally. The whole structure possibly represents a modified version of the subglenoid ridge seen in *Maj*. *crenatissimus*^75^, with its craniodorsal part representing the biceps tubercle^76^. Indeed, as already mentioned, that part of the bone is particularly expanded laterally, with the ventrally tapering surface medial to the groove representing the subglenoid fossa^75^. Such a groove is, to our knowledge, unknown in any related dinosaur and potentially represents an autapomorphy of *Ves*. *paranaensis*.

#### Forelimb

A left humerus (MPCO.V 0006d; Fig. 6e–j) and a significantly shorter long bone (MPCO.V 0006j; Fig. 6k–p), approximately half the length of the humerus, were found associated with the scapulocoracoid fragment MPCO.V 0006f. Based on the proportions seen in *Li*. *inextricabilis* (IVPP V15923), the second long bone fits the size expected for an antebrachial element associated with the humerus and is here interpreted as a left radius. A partial right hand (MPCO.V 0064b; Fig. 6q–r), with some articulated phalanges in very close association, has also been recovered from the sample. Taken together, the forelimb elements of *Ves*. *paranaensis* are not stout as those of abelisaurids^13,75^.

The humerus is a nearly straight bone, slightly bent caudally and medially within its proximal third. This differs from the laterally arching element of *Mas*. *knopfleri*^3^, and is more similar to the condition seen in *Li*. *inextricabilis*^57^. The proximal and distal ends are not expanded craniocaudally relative to the midline breadth of the shaft, but are lateromedially expanded to about 2.5 (proximal articulation) and 2.0 (distal articulation) times the minimal transverse width of the shaft. These expansions occur along the same plane, so that the long axes of both articulations are nearly aligned. This results in the non-twisted humeral shaft, typical of noasaurids^4^. However, the proximal expansion is somewhat rotated (ca. 15° clock-wise) in relation to the distal, as seen in proximal view. The humeral head has a rounded cranial/caudal profile, with an elliptical proximal outline that is slightly flattened caudally. This differs from both the more globular humeral head of abelisaurids^4,75,77^ and the less than two times wider than long humeral head of *Mas*. *knopfleri*^2^ and *El*. *bambergi*^4^. As in *Mas*. *knopfleri*^2^, the perimeter of the head is depressed and formed of more spongy bone, possibly representing the attachment of the acrocoracohumeral ligament^77^. The proximal expansion of the head is slightly medially displaced, leaving a lateral space proximal to the greater tubercle^2–4,73^. The greater tubercle expands craniolaterally as a rounded element, forming an angle of about 40° to the long axis of the head. It is distally placed and positioned at about the same level as the internal tuberosity, a condition considered synapomorphic for abelisauroids^77^. As in most noasaurids^2,4,57^, the internal tuberosity is highly reduced compared to that of other abelisauroids^4,75,78^. It corresponds to the pinched medial margin of the bone, as seen in proximal view. In cranial/caudal views, it forms a weak medial inflection on the proximalmost portion of the medial margin of the bone.

The deltopectoral crest starts as a faint ridge immediately distal to the greater tubercle. It expands distally, along the lateral margin of the humerus, reaching the middle of its proximodistal length and forming a 90° angle relative to the long axis of the distal condyles. In general form, the deltopectoral crest is a lateromedially compressed lamina that, in cranial view, arches laterally at its mid-length. This arched portion is also the most cranially projected (i.e. the apex of the deltopectoral crest), whereupon the crest corresponds to about 1/3 of the craniocaudal width of the humeral shaft. At this point, the crest has a slightly flattened cranial margin, merging smoothly into the shaft both proximally and distally. Similarly unexpanded deltopectoral crests are typical of abelisauroids^4^. The cranial surface of the humerus is smoothly depressed medial to the deltopectoral crest for the insertion of M. coracobrachialis^76^. Likewise, the lateral surface of the crest is also depressed proximal to its point of maximal inflection, probably for the insertion of M. deltoideous scapularis^74^. Caudal to that, a straight, rugose ridge extends from the lateral margin of the greater tubercle distally along the caudolateral corner of the bone, possibly representing the insertion area of M. latissimus dorsi^75^. Its proximal end is in the same position as the caudolateral tubercle of *E*. *bambergi*, considered to be an abelisauroid synapomorphy^4^. *Vespersaurus paranaensis* also lacks the medially broad caudal tuberosity reported in a Late Cretaceous Argentine abelisauroid^77^. A small foramen is visible on the craniomedial corner of the shaft, immediately opposite to the distal end of the deltopectoral crest, as also seen in *Mas*. *knopfleri*^3^.

The distal part of the humerus expands smoothly both laterally and medially. This gives rise to convex (in cranial/caudal views), but not prominent, radial and ulnar condyles. Accordingly, as also occurs in *Eo*. *mefi*^13^, the proximodistal flattening of the condyles, typical of ceratosaurs^3,4^, is absent in *Ves*. *paranaensis*. The distal condyles are separated caudally, distally, and cranially by a continuous shallow depression. This is most prominent on the cranial surface, where it forms a low, but well delimited semi-circular brachial fossa, which is absent in *El*. *bambergi* (Museum für Naturkunde, Berlin, Germany; MB R 4960) and *Mas*. *knopfleri*^3^. Medial to this fossa, a longitudinal ridge (“entr” in Fig. 7g) extends along the entepicondyle, fading proximally along the humeral shaft. The ectepicondyle, on the other hand, is laterally smooth, lacking any marked structure. The ulnar condyle is more craniocaudally expanded than the radial condyle, bearing a more rounded distal outline.

The associated left radius (MPCO.V 0006j) of *Ves*. *paranaensis* is a slender bone, almost ten times longer than minimally broad at mid-shaft. This differs from the stouter element of abelisaurids^75,78^ and even other ceratosaurs^13,79^, approaching instead the condition of *Li*. *inextricabilis*^57^. The radius represents ca. 47% of the length of the humerus, resembling the ratio present in *Eo*. *mefi* (49%^13^) and *Li*. *inextricabilis* (53%, IVPP V15923). The radius is slightly arched both medially and caudally, although this morphology is enhanced by its expanded proximal and distal articulations and by the longitudinally concave cranial and lateral margins. On the contrary, the caudal and medial margins are nearly straight. The proximal outline is eye-shaped, with the long axis almost twice broader than the short axis, and the caudal portion more proximally expanded. The distal articulation has an ovoid outline, with the medial margin representing the distal articulation of the ulna. Accordingly, the medial surface of the radius is covered by longitudinal striations immediately proximal to its distal articulation and its distal half is traversed by a strong longitudinal ridge (“drr” in Fig. 7l,o), which marks the caudal margin of the ulnar articulation. The articulation facets for the ulna at the distal and proximal portions of the radius are not in the same plane. As such, the radius is twisted (about 25°) along its shaft, although its “in life” position relative to the orthogonal planes is difficult to confidently infer. In any case, the part of the distal articulation opposite to the ulnar facet is more distally expanded, as also seen in various other theropods^80,81^.

Three digits are preserved in the recovered right hand (MPCO.V 0064b; Fig. 7q–r). The ungual phalanx and its proximally articulating element were preserved for both medialmost digits, whereas three bones were preserved in the lateralmost digit, including what is interpreted to be the terminal phalanx (see below). As no averostran theropod^82,83^ is known to possess more than two bones forming the fourth manual digit, we interpret the preserved digits as I−III. As such, regardless of its poor preservation, the presence of a biconcave proximal ginglymoid articulation indicates that the penultimate element preserved in digit I is a phalanx, not a metacarpal as in deeply-nested abelisaurids^75^. Its proximal articulation is much more expanded, both lateromedially and on the extensor-flexor axis, than the distal articulation, and bears a subtle extensor tuber. This expansion is more marked towards the flexor side, as seen in isolated noasaurid manual phalanges^3,84^. The bone is also only about 1.5 times longer than broad at its proximal end, departing from the plesiomorphically more elongated first phalanx of digit I seen in non-abelisauroid taxa, such as *Di*. *wetherilli*^70^, but apparently also in *Eo*. *mefi*^13^. Further comparisons to noasaurids and abelisaurids are hampered by the highly modified manus of the latter group^75^ and the lack of well-preserved hands in the former. The medial condyle of the distal articulation of the first phalanx is broken away, although it can be inferred that the whole articulation was flattened on the extensor-flexor axis. The bone is shorter than its associated ungual, which bears a well-defined extensor tuber, and both dorsal and ventral “blood grooves” in its medial surface (the former also seen in the exposed portion of the lateral surface), as occurs in the preserved manual ungual of *Mas*. *knopfleri*^2^. The ungual is not lateromedially flattened at its proximal portion, which tapper in all dimensions. It bears a distinctive, but minor ventral curvature, as reported for *Mas*. *knopfleri*^2^, and is also slightly curved medially.

The ungual and its previous phalanx are preserved in manual digit two. These are respectively exposed mainly in lateral and dorsal views, precluding assessment of many details. The non-ungual phalanx is only slightly longer than lateromedially broad at its proximal end, which is also only slightly broader than the distal end and bears a subtle extensor tuber. The distal articulation forms a symmetric ginglymus, with rounded condyles separated by a shallow extensor fossa, and a collateral pit is seen on the lateral side (the medial surface is covered by sediment). Although uncommon among averostrans, a non-elongated penultimate phalanx was also reported for *Li*. *inextricabilis*^57^. As in that taxon, the ungual of the second digit is longer than the preceding phalanx. Its biconcave proximal articulation is broader lateromedially than dorsoventrally deep. The bone is less recurved than that of the first digit, not nearly approaching the curvature of the possible second or third manual ungual of *N*. *leali*^84^. It bears a clear, low extensor tuber and a single (ventral) “blood” groove, related to the sheath cover, extending along the ventral margin of its lateral surface. This configuration suggests that *Ves*. *paranaensis* bore the generally stout/broad non-terminal phalanges of abelisauroids^4^ at least in digits I and II.

The more proximal element preserved in digit III has a planar proximal surface, with a broader than deep subtriangular outline (deeper medially and laterally ponied). As such, it is interpreted as a metacarpal. The bone is twice longer than broad at mid-length, with the medial distal condyle slightly more expanded distally than the lateral. The first phalanx is not block-shaped as in most abelisauroids^13,57^, being longer than twice its midline breadth. The terminal phalanx has a subtriangular proximal outline and is distally pinched, but not recurved, differing markedly from the larger recurved digit III ungual of *Li*. *inextricabilis*^57^. As such, although it retains a relatively elongated first phalanx, digit III of *Ves*. *paranaensis* seems to present a reduction in the number of phalanges comparable only to that of carnotaurines^75^ among abelisauroids. Obviously, the incompleteness of the *Ves*. *paranaensis* hand and the scarcity of information for most abelisauroid hands, coupled with homology uncertainties, precludes the establishment of robust evolutionary patterns.

#### Pelvic girdle

**Figure 8:**
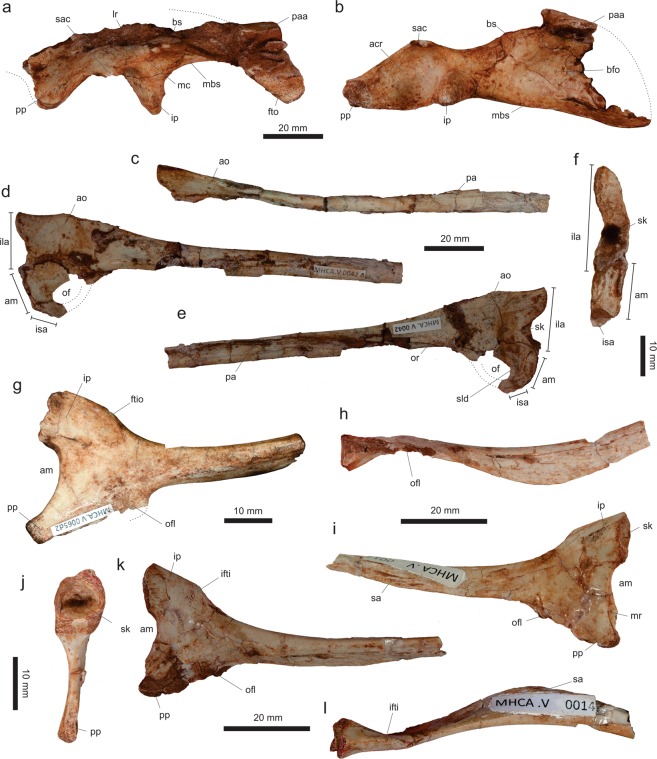
Pelvic girdle elements of *Vespersaurus paranaensis* gen. et sp. nov. (**a**,**b**) Left ilium (MPCO.V 0065d11, holotype) in lateral (**a**) and ventral (**b**) views. (**c**–**f**) Right pubis (MPCO.V 0042) in dorsal (**c**), lateral (**d**), medial (**e**), and proximal (**f**) views. (**g**) Left ischium (MPCO.V 0065d2; holotype) in lateral view. (**h**–**l**) Left ischium (MPCO.V 0014) in ventral (**h**), medial (**i**), proximal (**j**), lateral (**k**), and dorsal (**l**) views. Anatomical abbreviations: acr, acetabular roof; am, acetabular margin; ai, acetabular insisure; ao, M. ambiens origin; bfo, brevis fossa; bs, brevis shelf; ftio, M. flexor tibialis internus origin; fto, M. flexor tibialis musculature origin; ila, iliac articulation; ip, iliac peduncle; isa, ischiadic articulation; ip, ischiadic peduncle; lr, lateral ridge; mbs, “medial brevis shelf”; mc, medial ridge; of, obturator foramen; ofl, obturator flange; or, obturator ridge; pa, pubic apron; paa, postacetabular ala; pp, pubic peduncle; sac, supracetabular crest; sa, symphyseal articulation; sk, socket; sld, semi-lunate depression. Dashed lines represent reconstructed margins.

The holotypic left ilium MPCO.V 0065d11 (Fig. 8a–b) is partially preserved, lacking the entire preacetabular ala and the dorsal lamina, including the part that extended over the postacetabular ala. The latter ala is better preserved, but lacks most of its caudal margin, as well as the bone cover of the dorsal surface. It is strongly expanded lateromedially towards its caudal end, with a maximal breadth nearly twice that of the bone at the level of the acetabulum, resembling the condition in *Mas*. *knopfleri*^3^. This expansion is more marked towards the lateral than the medial side, so that the medial margins of the postacetabular ala and the iliac body form a low angle (about 165°) in ventral view. The ventral surface of the ala is entirely occupied by a deep funnel-shaped brevis fossa. This is bound medially by the sub-vertical ventral margin of the ala (=“medial brevis shelf”^3^) and laterally by a well-developed brevis shelf *sensu stricto*. As in *Mas*. *knopfleri*^3^, the brevis shelf extends ventrally, especially at its caudal end, as to almost entirely overhang and obscure the ventral margin of the ala (except for its cranioventral corner, just caudodorsal to the ischiadic peduncle) in lateral view. Although possibly exaggerated by compressive deformation, such a morphology seems to represent a further development, seen in both noasaurines^3^ and *G*. *sisteronis*^85^, from the typical condition present in other abelisauroids^4,86–88^. In any case, the lateral expansion of the postacetabular ala/brevis shelf has been considered a noasaurid synapomorphy^4^. In *Ves*. *paranaensis*, the caudal end of the brevis shelf gives rise to two cranially extending ridges. The more lateral of these (“lr” in Fig. 8a) forms a dorsal arch that nearly parallels the horizontal plane and is continuous to the supraacetabular crest. Such a connection between the brevis shelf and the supraacetabular crest via a strong ridge is typical of some early neotheropods and ceratosaurs^3,79^. The more medial ridge (“mc” in Fig. 8a) is much subtler, extending caudomedially and slightly ventrally from the brevis shelf, forming a craniolaterally bowing arch that delimits the craniolateral boundary of the brevis fossa. The caudolateral portion of the dorsal surface of the brevis shelf is covered by longitudinal scars, possibly representing the origin of the flexor tibialis musculature.

The iliac body is mostly preserved, including the supraacetabular crest, acetabular wall, as well as the pubic and ischiadic peduncles, although the distalmost end of the pubic peduncle is damaged. The acetabulum is fully open, with vestiges of its medial wall only at the craniodorsal corner. The supraacetabular crest is well-developed and ventrally overhanging, so that the dorsal margin of the acetabular opening is hidden in lateral view, resembling the condition in *Mas*. *knopfleri*^3^. The dorsal/ventral outline of the supraacetabular crest of *Ves*. *paranaensis* is subtriangular, with its rounded lateral terminus positioned within the caudal half of the acetabulum, as in *Mas*. *knopfleri*^3^, where it represents about half the lateromedial breadth of the bone. In contrast, the same profile is subrectangular in *El*. *bambergi* (MB R 4960). From the point of maximal lateral expansion, the caudal continuation of the crest becomes abruptly less laterally projected, forming the caudodorsal corner of the acetabulum and bifurcates into two lower ridges. The most conspicuous of these ridges extends caudally to join the brevis shelf, whereas a much subtler ventral extension forms the medial margin of the caudal surface of the acetabulum. Cranial to the point of maximal lateral projection, the supracetabular crest continues as a laterally expansive and ventrally overhanging flange. It bows dorsally, following the craniodorsal contour of the acetabulum, and extends along the pubic peduncle, where it merges smoothly to its lateral surface. The acetabular roof is also exposed in ventral view in another specimen of *Ves*. *paranaensis* (MPCO.V 0064a), along with a partial sacral series. The exposed portion of this ilium matches the previously described bone in all details, demonstrating that the lateromedial breadth of the acetabulum is greater than half the space between the iliac pair when they are in articulation.

Unlike *Mas*. *knopfleri*^3^ and various abelisaurids^13^, *Ves*. *paranaensis* does not have an ischial peduncle that, relative to the pubic peduncle, projects significantly ventrally. However, as is typical of abelisauroids^12^, the distal articulation of both peduncles display the “peg” morphology whereby they would have locked into their respective sockets on the proximal pubis and ischium. Although its cranial surface is broken, it is possible to infer that the pubic peduncle had a subtriangular ventral outline, with lateral, craniomedial, and caudal margins. The latter forms the base of the cranial surface of the acetabulum, between the caudomedial and caudolateral corners, with the “peg” located at the craniomedial portion of the articulation. The former of those corners is continuous with a ridge that extends caudodorsally, forming the sharp medial margin of the cranial surface of the acetabulum. The caudolateral of those corners is caudoventral to the cranioventral tip of the supraacetabular crest, which does not reach the articulation facet itself. The better preserved ischiadic peduncle is also subtriangular in distal outline, with a pointed lateral corner and a convex medial margin. The “peg” is positioned in the centre of the articulation, displaced towards its medial side and surrounded by portions of the articulation that are not as ventrally extensive. This mirrors the ischial “socket”, which is also slightly displaced medially on its articular surface.

The preserved medial surface of the ilium is mostly flat, with shallow depressed areas above the peduncles and a more medially expanded area dorsal to the acetabular aperture. An ovoid depression dorsal to the ischiadic peduncle and below an oblique shelf that extends along the postacetabular ala possibly represents the fourth sacral rib articulation, as is seen in *Mas*. *knopfleri*^3^. Likewise, another oblique shelf that follows the dorsal contour of the pubic peduncle probably represents the dorsal margin of the more craniocaudally extensive articulation of the second sacral rib.

The isolated right pubis MPCO.V 0042 (Fig. 8c–f) preserves most of the body and about the proximal three-fourths of the shaft. The body is dorsoventrally expanded and lateromedially compressed. Moving dorsally to ventrally in lateral view, its proximal articulation includes a sigmoid (convex at the dorsal third and concave ventral to that) iliac articulation, the acetabular margin, and the ischiadic articulation, the latter two of which are straight and form an angle of 120° to one another. These elements are enhanced in medial view, in which two markedly convex margins meet at a nearly right angle, directly medial to a deep excavation on the articular surface. The dorsal of these convex margins represents the dorsal two-thirds of the iliac articulation, whereas the lower encompasses the lower part of that articulation, as well as the acetabular margin and the ischiadic articulation. In proximal view, the iliac articulation arches laterally around the deep medial excavation, dorsal to which the articular facet is dorsoventrally convex. This morphology matches the “peg-and-socket”^12^ arrangement seen in *Mas*. *knopfleri*^2^, which also bears a deep, medially displaced socket for the iliac articulation. The entire proximal articulation area of the pubis is, however, more lateromedial compressed in *Ves*. *paranaensis* than in *Mas*. *knopfleri*^2^.

The dorsal margin of the pubic body of *Ves*. *paranaensis* is marked by a subtle angular expansion. This, along with a depressed area positioned proximoventrally from it, on the lateral surface of the bone, represents the origin of M. ambiens^89^. This contrasts with the origin of this muscle in *Mas*. *knopfleri*^2^, which is mostly represented by the depressed area on the lateral surface of the bone. The obturator plate is incomplete ventrodistally, but it is possible to infer a rounded profile and the presence of single a suboval obturator foramen, as occurs in *Mas*. *knopfleri*^2^, but not in *Li*. *inextricabilis*, which possesses two apertures in the obturator plate^57^. In medial view, the proximal and dorsal margins of the foramen in *Ves*. *paranaensis* are flanked by a semi-lunate depression (“sld” in Fig. 8e), which extends proximally to form deep pocket within the pobic body. In contrast, that surface bears a proximodistally elongated groove in *Mas*. *knopfleri*^3^. The obturator plate extends distally as a ridge (“or” in Fig. 8e) along the ventral portion of the medial surface of the shaft. Dorsal to that ridge, the proximal part of the shaft bears a subtle longitudinal groove, which extends as a shallow fossa on the medial surface of the pubic body, dorsal to the obturator plate. More distally along the shaft, that medial ridge is more expanded, forming the laminar medial contact of the pubic pair, i.e. “pubic apron”^2^. As a result, the pubic shaft has a “crescent-shaped” cross section (with a ventromedially facing concavity) at its more distally preserved portion, composed of a dorsoventrally deeper lateral rod-like body and a more dorsoventrally flattened portion extending medially from its dorsal portion. In general, the preserved portion of the pubic shaft is relatively straight, in both lateral/medial and dorsal-ventral views.

The partial left ischia MPCO.V 0065d2 and MPCO.V 0014 (Fig. 7g–l) resemble one another in most details. Both lack the distal half of the shaft as well as the ventral margin of the obturator flange and the proximal margin of the pubic peduncle. Their proximal bodies are composed of well-developed iliac and pubic peduncles, separated by a deeply incised acetabular margin that bows slightly laterally in proximal view. The pubic peduncle is longer, but not as dorsoventrally deep as the iliac peduncle. The latter is also much more lateromedially expanded than both the pubic peduncle and the more laminar portion of the bone that extends ventrally from it. The proximal ends of both peduncles have rugose and striated outer margins, indicating the presence of ligamentous attachments. As seen in MPCO.V 0014, the proximal surface of the iliac articulation has a rounded outline and is deeply excavated along its entire surface, forming the socket of the “peg-and-socket” articulation typical of abelisauroids^12^. The margins around the socket are mostly eroded in MPCO.V 0014, but MPCO.V 0065d2 shows that they expand proximally, especially at the dorsal and ventral parts of the articulation, which acquires a concave lateral profile. The most conspicuous feature of the iliac peduncle is the rugose origin point of M. flexor tibialis internus^3,89,90^, situated laterally on its dorsodistal portion. This is better developed than that of *Mas*. *knopfleri*^3^, forming a projection. The tip of that projection forms a low angle between the straight dorsal margin of the iliac peduncle and the concave margin of the bone distal to that. As such, the proximal portion of the iliac peduncle has a slight ventral lean (relative to the long axis of the bone in lateral view), as is also manifested by the dorsal margin of the ischial acetabulum. Indeed, as in *Mas*. *knopfleri*^3^, this does not follow the rounded contour of the rest of its margin, but is also ventrally leaned. The ventral margin of the pubic peduncle extends distally to form a low obturator flange. As is typical of noasaurids, that flange lacks a distal notch separating it from the ischial shaft. However, a subtle distal incision is present (Fig. 8g) and the flange does not smoothly merge with the shaft as in *Li*. *inextricabilis*^57^ and *E*. *bambergi*^4^. Likewise, a secondary notch, as seen in *Mas*. *knopfleri*^3^, is also lacking. The medial surface of the ischial body is mostly concave, with a ridge (“mr” in Fig. 8i) flanking the ventral half of the acetabular margin. In ventral/dorsal views, the preserved part of the ischium is sigmoid, arching laterally at its proximal portion and medially along the proximal part of the shaft. The latter also arches slightly ventrally in lateral view. Immediately distal to the obturator flange, the ischial shaft has a dorsoventrally elongated cross section. Yet, as it extends distally, the ventral portion of the shaft twists medially, so that the cross section becomes lateromedially elongated, although a laminar medial flange is lacking. Instead, the symphyseal medial articulation to the antimere is deep and striated, marking the distal half of the persevered part of the shaft. The symphyseal articulation occupies the entire dorsoventral depth of the shaft more distally, but tapers to its ventral margin as it extends proximally. The cross section of the most distally preserved part of the shaft is ovoid, with a lateromedial long axis.

#### Tibia

**Figure 9:**
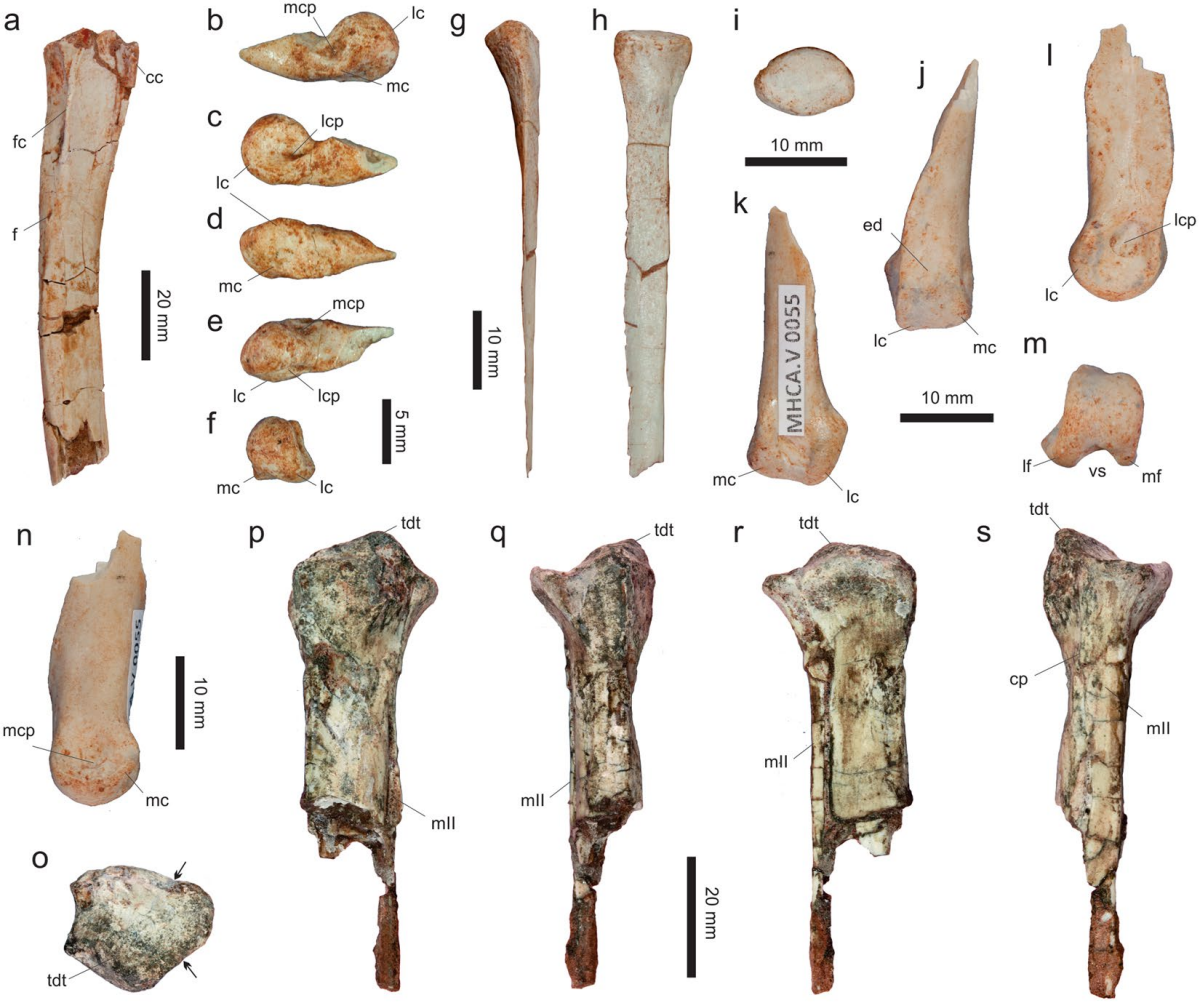
Tibia, tarsus, and metatarsus of *Vespersaurus paranaensis* gen. et sp. nov. (**a**) Right tibial shaft (MPCO.V 0018a-c) in lateral view. (**b**–**f**) Left metatarsal I (MPCO.V 0057b) in medial (**b**), lateral (**c**), ventral (**d**), dorsal (**e**), and proximal (**f**) views. (**g**–**i**) Right metatarsal II (MPCO.V 0063a) in caudal (**g**), lateral (**h**), and proximal (**i**) views. (**j**–**n**) Right metatarsal II (MPCO.V 0055) in dorsal (**j**), ventral (**k**), lateral (**l**), distal (**m**), and medial (**n**) views. (**o**–**s**) Left third distal tarsal and metatarsals II-III (MPCO.V 0016) in proximal (**o**), caudal (**p**), lateral (**q**), cranial (**r**), and medial (**s**) views. Anatomical abbreviations: cc, cnemial crest; cp, caudal projection; ed, extensor depression; f, foramen; fc, fibular crest; lc, lateral condyle; lcp, lateral collateral pit; lf, lateral flange; MII, metatarsal II; mc, medial condyle; mcp, medial collateral pit; mf, medial flange; tdt, third distal tarsal; vs, ventral sulcus. Arrows indicate the contact between metatarsals II and II in proximal view.

A single right tibial shaft (MPCO.V 0018; Fig. 9a), lacking both extremities was preserved in the sample. It is caudally bowing in lateral/medal views, and sigmoid in cranial/caudal views, with a slight medial bowing proximally and a lateral deflection more distally. The mid-shaft cross-section is ovoid, with a craniomedially to caudolaterally oriented long axis and a flattened craniolateral margin. More proximally, however, due to the cranial expansion of the cnemial crest, the tibial cross section is subtriangular. Its most conspicuous feature is the sharp fibular crest, which extends along the lateral surface of the proximal part of the shaft, turning slightly caudally at its distal end. Contrasting with *Mas*. *knopfleri*^3^, the crest does not extend distally to form a marked fibular flange. The fibular crest forms the boundary between the lateral fossa^3,8^, cranially, and another depression located on the caudolateral surface of the bone. The latter is caudally bounded by a fainter ridge, with a nutrient foramen piercing the tibial shaft just distal to that depression, as also seen in *Mas*. *knopfleri*^3^. The lateral fossa^3,8^ is well developed and the distal end of the cnemial crest expands slightly laterally along its cranial border. Cranial and lateral longitudinal intermuscular lines extend distally from the cnemial and fibular crests respectively.

#### Tarsus

A left third distal tarsal (Fig. 9o–s) is the only tarsal element known for *Ves*. *paranaensis* and was preserved along with the articulated proximal portions of metatarsals II−III (MPCO.V 0016). It covers the caudolateral half of the proximal articular surface of metatarsal III and is partially co-ossified to it, as occurs in the relatively advanced ontogenetic stages of several early neotheropod and coelophysoid species^91,92^. Overall, the proximal surface of the tarsal is convex, with its proximal apex placed near its medial margin, close to the articulation for metatarsal II. From that point, the proximal surface descends more abruptly towards its medial edge, forming a longer and more gently sloping surface towards its lateral margin. The proximodistal width of the bone at its apex is about the same as its minimal breadth as seen in proximal view. In the same view, the tarsal is caudomedially to craniolaterally elongated, being twice as long along that axis than on the opposite one.

#### Pes

**Figure 10:**
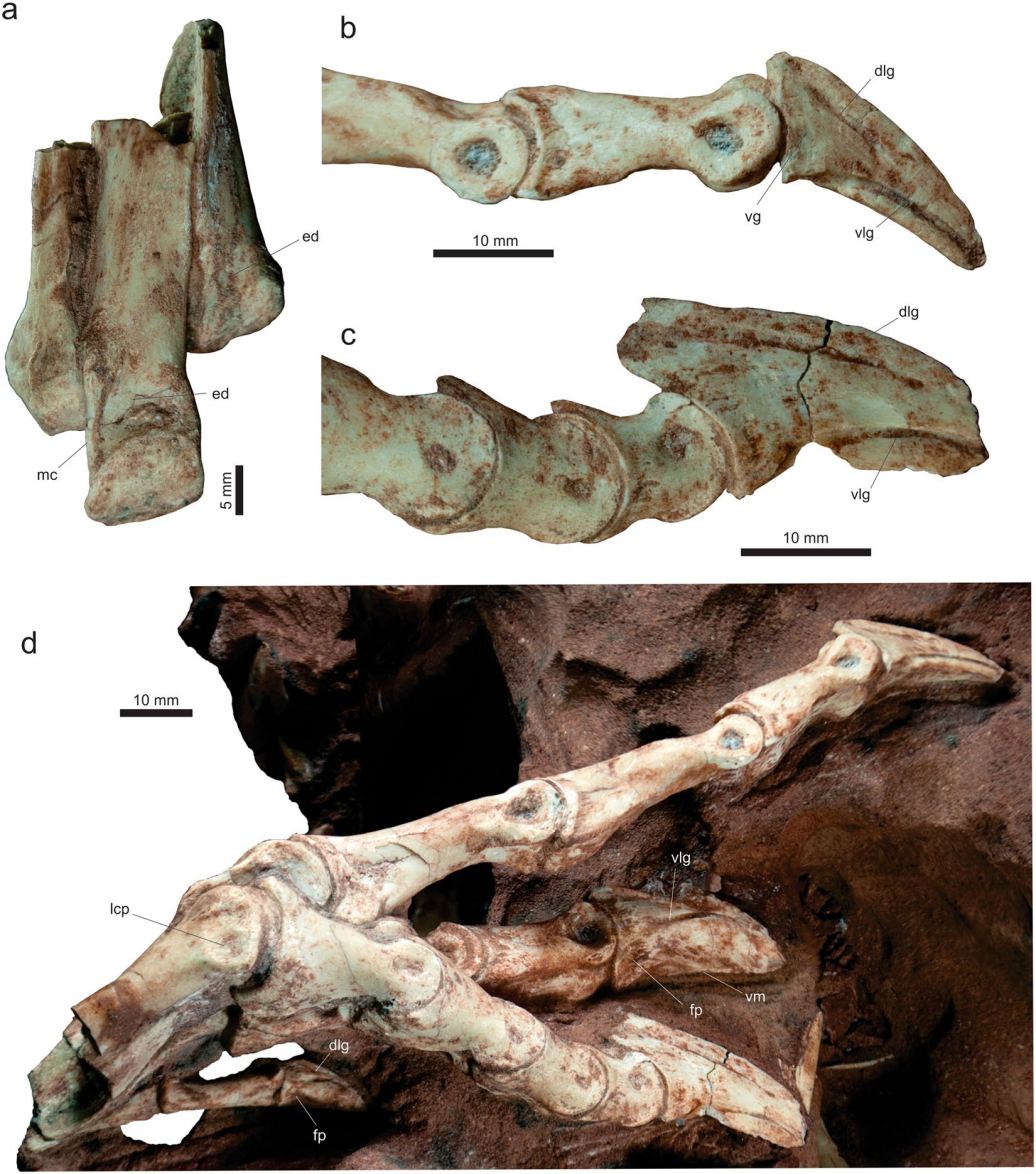
Holotype right pes of *Vespersaurus paranaensis* gen. et sp. nov. (MPCO.V 0065d1). (**a**) Distal end of metatarsal II-IV in dorsal view. (**b**) Distal part of digit II in lateral view. (**c**) Distal part of digit IV in lateral view. (**d**) Entire foot as preserved in lateral view. Anatomical abbreviations: ed, extensor depression; fd, flexor depression; lcp, lateral collateral pit; mc, lateral crest; plg, proximolateral groove; vg, vertical groove; vlg, ventral lateral groove; vm, ventral margin.

The metatarsus of *Ves*. *paranaensis* is known based on a complete metatarsal I, which is articulated to the distal portions of metatarsals II−IV of the holotypic right foot (MPCO.V 0065d1; Fig. 10), an isolated left metatarsal I (MPCO.V 0057b; Fig. 9b–f), an isolated right metatarsal II lacking its distal part (MPCO.V 0063a; Fig. 9g–i), the isolated distal end of another right metatarsal II (MPCO.V 0055; Fig. 9j–n), and the articulated proximal parts of left metatarsals II−III (MPCO.V 0016; Fig. 9o–s). The holotypic metatarsal set (MPCO.V 0065d1) was originally more complete, but the more proximal portions were lost prior to the current description. Nonetheless, older photographs of these elements exist (see Supporting Information), and these help to elaborate some of the pedal anatomical traits of *Ves*. *paranaensis*. Compared to *Vel*. *unicus*^7^ and based on the length of the phalanges and the lateromedial breadth of the distal end of the third metatarsal, we estimate that slightly more than one third of the metatarsus is currently preserved in the holotype, but that at least three quarters were originally preserved.

Metatarsal I is a small bone, 18 mm long in the holotype (Fig. 10d), with a partial left isolated element (Fig. 9b–f) measuring 13 mm long as preserved. As seen in the holotype, the bone was appressed to the medial surface of the distal portion of metatarsal II, its distal end positioned four centimetres proximally from that of its laterally neighbouring metatarsal. The lateral margin of the bone is flattened, matching the overall lateromedial compression of its wedge-shaped shaft. Its enlarged distal portion forms well-developed condyles, the lateral of which is more distally and craniocaudally expanded than the medial, with a predominant cranial projection. It bears a deep collateral ligament pit, the caudal margin of which is formed by a curved ridge that extends proximally to form the craniolateral corner of the shaft. The medial condyle is craiocaudally depressed and the surface housing its shallow collateral pit faces craniomedially. The caudal margin of that condyle forms the pointed medialmost extension of the distal articulation. The metatarsal has a subtriangular distal outline, with a flattened caudal margin. This margin is caudolaterally oriented in the articulated pes of the holotype, so that the digit extends somehow behind the rest of the foot.

Based on photographs of the holotype when it was more complete (see Supporting Information), the shaft of metatarsal II is strongly compressed lateromedially. Its midshaft transverse breadth was less than one fifth that of metatarsal III, a condition confirmed by MPCO.V 0016 (Fig. 9p–s). In that specimen, the proximal part of the metatarsal II shaft has a cross section slightly less than three times broader craniocaudally than lateromedially. Poor preservation hampers a more precise estimate of this ratio in the more distal parts of the shaft. On the contrary, the best preserved MPCO.V 0063a (Fig. 9g–i) has a laminar shaft that is lateromedially narrower than one fifth of its craniocaudal width. The lateral surface of its shaft is longitudinally traversed by a broad/shallow groove, imparting a subtle craniocaudal concavity. This indicates a closely appressed articulation with metatarsal III. The cranial part of the shaft is lateromedially broader, with a transversely rounded cranial margin, whereas the caudal part is more strictly laminar (Fig. 9g), tapering to a sharp margin. Estimated from the craniocaudal length of its proximal articulation and based on closely related taxa^4,7^, MPCO.V 0063a preserves roughly the proximal three-quarters of the bone, indicating that metatarsal II is lateromedially flattened along most of its shaft, as confirmed by the compression of the proximal-most preserved portions of the bone in both MPCO.V 0065d1 and 0055. *Vel*. *unicus*^8^, *Mas*. *knopfleri*^2^, and *N*. *leali*^5^ share generally similar shaft proportions of the metatarsal II. Indeed, a lateromedial compression of metatarsal II was considered synapomorphic for the clade formed of Elaphrosaurinae + Noasaurinae^4^, although this trait is clearly more marked in core-noasaurines than in *El*. *bambergi*^4,12^ or *Li*. *inextricabilis* (IVPP V15923). However, the metatarsal II of *Ves*. *paranaensis* is even more laminar than those of all other noasaurines.

The proximal articulation of metatarsal II (Fig. 9i,o) expands strongly craniocaudally and especially lateromedially from its shaft. Although the articulation with metatarsal III is not clear in the proximal view of MPCO.V 0016, it is well marked both cranially and caudally (arrows in Fig. 9o). It is, therefore, possible to infer an ovoid proximal outline, that was slightly elongated craniocaudally. The articular face has a slight proximal expansion within its caudal half, although the surface is subtly concave overall. The proximal articulation of the bone is positioned somewhat displaced cranially with respect to the lateral surface of metatarsal III, as is also seen in *El*. *bambergi*^4^. In medial view, it is clear that the proximal expansion is particularly marked towards its cranial surface, whereas its medial surface is marked by longitudinal striations. The caudal margin merges more subtly into the shaft, although it bears a caudally angled projection (“cp” in Fig. 9s). The proximal outline of MPCO.V 0063a is also ovoid, one third craniocaudally longer than lateromedially broad. Its medial margin is rounded, whereas the lateral is flattened for articulation with metatarsal III, with a subtle lateral inflection point. The “reduction of the proximal end of metatarsal II” has thus far been the only unambiguous synapomorphy proposed for Noasaurinae^4^. Indeed, compared to that of metatarsal III, the proximal end of metatarsal II of *Ves*. *paranaensis* is reduced relative to that of *El*. *bambergi*^4^ and *Li*. *inextricabilis* (IVPP V15923), resembling the conditions in *Mas*. *knopfleri*^2^ and *Vel*. *unicus*^7,8^. Furthermore, based on comparisons to its distal articulation, the proximal end of metatarsal II seems even less expanded in *N*. *leali*^5^ than in the above taxa.

The distal end of metatarsal II (Figs 9k–n, 10a,d) is well expanded lateromedially from the laminar shaft, the distal part of which has a crescentic cross section; i.e. rounded medially and subtly concave laterally. As a result, the bone has sharp cranio- and caudolateral corners, an indication of its close contact to metatarsal III. The former corner has a craniolateral inflection also seen in *Mas*. *knopfleri*^2^, medial and distal to which the cranial surface of the bone is flattened, with a very subtle “hyperextension” depression^2^. Both collateral ligament pits are well-developed, but the lateral is significantly deeper. The distal outline is slightly craniocaudally deeper than lateromedially broad, with a caudal sulcus that is shallower than those of *Mas*. *knopfleri*^2^ and *N*. *leali* (PVL 4061), excavating about 30% of the distal depth of the bone. The medial flange is narrower than the lateral, but this discrepancy is less marked than in *N*. *leali* (PVL 4061) and *Mas*. *knopfleri*^2^. The caudal surface of the lateral flange is flattened, with a lateral expansion projecting below the collateral pit.

Metatarsal III (Figs 9o–s, 10a,d) is by far the most robust of the metatarsals, and likely the main weight-bearing metapodial element. Its proximal outline (Fig. 9o) is hourglass-shaped, well excavated both medially and laterally to accommodate the proximal ends of metatarsals II and IV, respectively. These excavations indicate that the proximal articulation of metatarsal II was significantly more pronounced than that of metatarsal IV. In proximal view, between the articulations for metatarsal II and IV, the cranial margin of metatarsal III is rounded, whereas the caudal margin is more pointed. The cranial part of the articular facet bears a broad transverse groove, forming a craniocaudally concave surface. Caudomedial to that, the facet is covered by the third distal tarsal. In lateral/medial views, following the pattern seen in metatarsal II, the proximal end of metatarsal III expands more abruptly towards the cranial side, whereas the caudal margin bears a caudal bulge, also seen in *Vel*. *unicus*^7,8^ and *Mas*. *knopfleri*^3^.

Breakages in the shaft of both metatarsals III of MPCO.V 0016 and 0065d1 reveal an ovoid cross-section for the former (with a lateromedial long axis) and a more rounded, but medially (towards metatarsal II) flattened, cross-section for the latter. The distal end of the bone (Fig. 10a) is kinked medially relative to the long axis of the shaft proximal to that, as also seen in other abelisauroids^86^. The cranial surface of this kinked portion bears a well delimited, drop-shaped “hyperextension” depression. This depression is bound laterally by a marked crest (“mc” in Fig. 10a), that is cranially expanded in its proximal portion, and distally by a very proximally extensive (much more than in the neighbouring metatarsals), semilunate phalangeal articulation. A subtler border marks the medial boundary of that depression. The distal outline of metatarsal III was subrectangular, slightly deeper than broad, with both collateral pits present and moderately deep. Further details of the distal end of metatarsal III are obscured by the articulation to the other metatarsals and the proximal phalanx of the pedal digit.

Old photographs of the holotype reveal that the shaft of metatarsal IV was strongly compressed lateromedially, about four times broader craniocaudally than lateromedially at midshaft. This differs from the uncompressed metatarsal of *Mas*. *knopfleri*^2^, as well as from the rod-like element of *Vel*. *unicus*^8^. However, the bone expands lateromedially as it extends distally; its shaft bearing an ovoid cross section at its current most proximally preserved portion (c. 6 mm broad lateromedially and 8 mm craniocaudally), with a flattened medial margin. Unlike in *Mas*. *knopfleri*^2^, this indicates a close adherence to metatarsal III continuing to the distal end of the bone. Such a morphology is seen in *Vel*. *unicus*^7,8^, albeit without the lateromedial compression of the shaft seen in *Ves*. *paranaensis*. The distal articulation of metatarsal IV is deeper craniocaudally than lateromedially broad, but is also lateromedially compressed at its cranial half. This is about 80% the breadth of the caudal margin, which displays a slight concavity between the distal condyles. This results in an asymmetric articulation, wherein the cranial surface faces craniolaterally, the lateral collateral pit faces slightly cranially and is caudally positioned on the lateral surface of the bone, a condition very similar to that of *Mas*. *knopfleri*^2^. In fact, the lateral surface of the distal end of the bone is excavated by two pits, caudoproximally and craniodistally placed and separated by an oblique ridge.

Based on its holotype (Fig. 10), the phalangeal formula of *Ves*. *paranaensis* for digits I−IV is 2-3-4-5, all of which bear an ungual terminal phalanx. Phalanx 1 of the first digit is elongated, nearly four times its distal breadth. The distal articulation is as dorsoventrally deep as lateromedially broad, whereas the proximal articulation is slightly deeper, with the shaft narrowing to a minimal breadth of about half of the dorsoventral depth. The ventral margin of the proximal portion bears a longitudinal ridge slightly displaced laterally. The distal condyles are raised higher towards the dorsal side, with a depressed area separating them ventrally. This represents a moderately developed flexor pit, whereas the collateral pit is well-developed on the lateral surface (the opposite being covered by sediment). The ungual is shorter than the preceding phalanx and, although narrowing towards its pointed proximal portion, is not trenchant. Its lateral surface bears an eye-shaped depression at its proximodorsal corner, which extends distally (“dlg” in Fig. 10d) following the contour of the dorsal margin of the bone. The distal part of this groove approaches a sigmoid (ventrally arching at its proximal part and dorsally arching at the distal part) groove extending along the lateral surface of the ungual. This set of grooves correspond to the “Y-shaped” system of lateral vascular grooves typical of abelisauroid theropods^93^. On the lateral surface, the area ventral to the lower groove is lateroventrally facing and aligned with the ventral surface of the previous phalanx. Therefore, although laterally exposed, this surface corresponds to the ventral surface of the ungual of theropods in general. This bears a proximal flexor pit (“fp” in Fig. 10d) and another groove extending distally from it. Indeed, because the margin separating the original (as generally seen in theropods) lateral and ventral surfaces of the ungual is dorsally positioned, the actual ventral margin of the ungual corresponds to the margin separating its original ventral surface from the medial surface of the bone. This margin is laminar, nearly straight in lateral/medial views, and bears a small proximal concavity.

The first phalanx is the longest of digit II. Its shaft is lateromedially compressed, and the distal articulation is deeper dorsoventrally than lateromedially broad. The extensor pit is also lateromedially compressed and the medial collateral pit is rounded and shallow. It bears a subtle extensor tuber at its proximal articulation, as also seen in the second phalanx. Its distal articulation with the latter is lateromedially compressed with a very subtle extensor pit and a broad and deep lateral collateral pit. The lateral distal condyle is raised dorsally relative to the medial, reflecting the anatomy of the ungual, as also seen (to a lesser degree) in digit I. Following that rise, the lateroventral corner of the proximal articulation of the ungual is also positioned more dorsally. As a result, the original (in the above sense) ventral surface of the bone is lateroventrally facing and its original medioventral margin functions as the laminar ventral margin of the ungual (“vm” in Fig. 10d). The original ventral surface of the ungual is traversed by a shallow longitudinal groove and its proximal portion bears a marked flexor pit. A pair of longitudinal grooves excavates the lateral surface of the ungual. The ventral groove (“vlg” in Fig. 10d) extends dorsodistally from the proximoventral portion of the bone to meet its dorsal counterpart, which follows the contour of the dorsal margin of the ungual at its proximal end, whereupon they extend distally as a subtler, conjoined groove that also follows the dorsal contour of the bone. The ungual is strongly flattened lateromedially and slightly longer than the second phalanx.

Phalanx 1 of digit III is the longest in the foot. Both its shaft and distal articulation are lateromedially compressed. The distal end has well-developed extensor and collateral pits, being also depressed ventrally between the condyles. The second phalanx is nearly as long as the first and similar to that in most morphological aspects, although it bears an extensor tuber, which is lacking in the preceding element. The ventral surface is flat and lateromedially expanded in the proximal portion of the bone, resulting in a subtriangular surface in plantar view. The shorter third phalanx has a better developed extensor tuber at the proximal articulation, a well-developed lateral collateral pit (the medial is covered by sediment), but no extensor pit. Only a longitudinal groove separates the distal condyles dorsally. In contrast to the condition in the second digit, the medial condyle is displaced dorsally relative to the lateral. The ungual is intermediate in length between phalanges 2 and 3, and slightly shorter than that of the second digit. It is less dorsoventrally expanded at its proximal portion and lateromedially compressed than the unguals of the adjacent digits. Its lateral surface is traversed by well-developed dorsal and ventral longitudinal grooves, which follow the contour of the respective margins of the bone. As such, the ungual of digit III lacks the ventral surface modifications seen in the other digits. In fact, the proximal end of the ventral longitudinal groove extends onto the ventral surface of the bone, where it is waisted in ventral view. A clear flexor pit excavates the ventral surface of the bone proximal to that, and its ventral margin is expanded lateromedially more proximally.

Phalanx 1 of digit IV is shorter than those of digits II and III and subequal to phalanx 3 of the previous digit, as seen in *Vel*. *unicus*^8^. It is much deeper dorsoventrally at its proximal than at its distal articulation. The former has well developed extensor and flexor tubera, the latter of which is rugose and ventrally flat. The distal end bears a faint lateral collateral pit. The shorter second phalanx resembles the former, but is not so dorsoventrally expanded at its proximal articulation. Both bear shallow extensor pits. Morphologically similar to the previous one, phalanx 3 is more compact, only slightly proximodistally longer than dorsoventrally deep. On the contrary, phalanx 4 is deeper than long and has a deeper and larger lateral collateral pit. The large ungual is the longest phalanx of the digit. In contrast, phalanx 1 is proportionally longer than the ungual in the fourth pedal digit of *Vel*. *unicus* (Museo de la Universidad Nacional del Comahue, Neuquén, Argentina; MUCPv 41).

As with the other phalanges of the holotype of *Ves*. *paranaensis* (Fig. 10), only the lateral surfaces of the unguals are exposed. None of them are strongly recurved, bearing a straight to subtly concave ventral margin. Except for the third ungual, they are distinctly more lateromedially compressed than the ungual reported for *Mas*. *knopfleri*^2^ and those of *Vel*. *unicus* (MUCPv 41). Ungual of digit II is relatively the deepest, with the dorsoventral depth of the proximal margin about two-thirds of the entire length of the bone. It has the “Y-shaped” system of lateral vascular grooves typical of abelisauroid theropods^93^, including noasaurines^2,6^. The surface between those dorsal and ventral branches is convex, but not protruding as in some abelisaurid unguals^93^. The ungual of digit III is the broadest element in the pes lateromedially, but the shallowest dorsoventrally, with a sharper distal tip and the depth of the proximal margin less than half the proximodistal length of the bone. This is heightened by a proximally-projecting, well-developed extensor tuber, with the proximal and dorsal margins of the bone forming an angle of about 120° (that angle is about 90° in the ungual of the second digit). Unlike the condition in the former ungual, the pair of lateral grooves does not merge distally. Instead, the ventral one extends along the entirety of the ventral margin of the ungual, whereas the dorsal is restricted to the proximal half of the dorsal margin, as also seen in some abelisauroid unguals^93^. Flanking its proximal articulation, the lateral surface of that phalanx bears the proximal groove (“vg” in Fig. 10d) seen in *Mas*. *knopfleri*^2^. The ungual of the fourth digit is the largest of the pes. There is a deep, well-developed extensor tuber and the lateral grooves are not distally connected. It is much deeper than the previous ungual, roughly twice as long proximodistally than dorsoventrally deep at its proximal end. It is interesting to note that the pedal ungual of digit IV of *Vel*. *unicus* (MUCPv 41) bears a closer resemblance to the ungual of its digit III, rather than to that of the digit IV of *Ves*. *paranaensis*.

**Figure 11:**
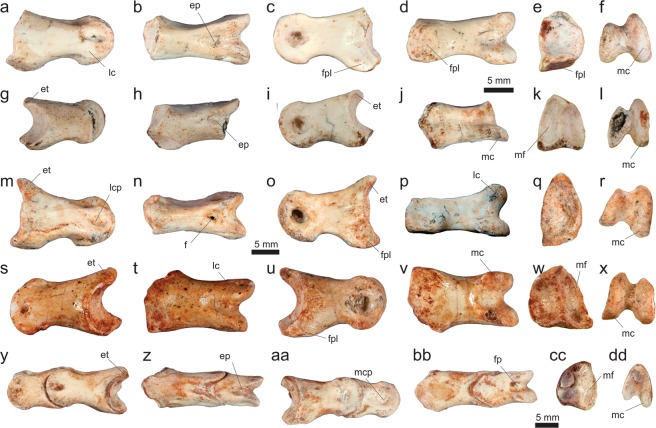
Non-ungual pedal phalanges of *Vespersaurus paranaensis* gen. et sp. nov. (**a**–**f**) Phalanx 1 of right digit IV (MPCO.V 0056a) in lateral (**a**), dorsal (**b**), medial (**c**), ventral (**d**), proximal (**e**), and distal (**f**) views. (**g**–**l**) Phalanx 2 of right digit IV (MPCO.V 0056b) in lateral (**g**), dorsal (**h**), medial (**i**), ventral (**j**), proximal (**k**), and distal (**l**) views. (**m**–**r**) Phalanx 1 of right digit IV (MPCO.V 0060) in lateral (**m**), dorsal (**n**), medial (**o**), ventral (**p**), proximal (**q**), and distal (**r**) views (**s**–**x**). Phalanx 2 of left digit IV (MPCO.V 0057a) in lateral (**s**), dorsal (**t**), medial (**u**), ventral (**v**), proximal (**w**), and distal (**x**) views. (**y**–**dd**) Phalanges 2 and 3 of a left digit IV (MPCO.V 0006k) in lateral (**y**), dorsal (**z**), medial (**aa**), ventral (**bb**), proximal (**cc**), and distal (**dd**) views. Anatomical abbreviations: ep, extensor pit; et, extensor tuber; f, foramen; fp, flexor pit; fpl, flexor platform; lc, lateral condyle; lcp, lateral collateral pit; mc, medial condyle; mcp, medial collateral pit; mf, medial facet.

A series of non-ungual pedal phalanges were found isolated. A pair or articulated elements (MPCO.V 0056a-b) represent phalanges 1–2 of a right digit IV. The former (Fig. 11a–f) has a concave, “D-shaped” (slightly deeper than lateromedially broad) proximal articulation, medially flattened where it bordered metatarsal III. The bone is about twice longer than dorsoventrally deep and lateromedially broad at its articulations. The proximal end is slight deeper and narrower than the distal end. The proximal articulation has an extensive ventral process. This is more marked towards the medial side, medially bounding a flat and rugose flexor platform. This platform faces slightly laterally, its more ventrally projecting medial half bearing a depressed surface. The distal ginglymus is asymmetric, with a dorsoventrally deeper and lateromedial broader medial condyle. These are separated dorsally, distally, and ventrally by a well-developed intercondylar groove. Its dorsal branch is continuous to a deep extensor pit with smooth margins. Both collateral pits are also well developed. The shaft has a dorsoventrally convex lateral surface, whereas the medial is flatter. Matching the preceding element, the proximal articulation of the second phalanx (Fig. 10g–l) is asymmetric, with the deeper kidney-shaped medial facet expanding dorsal to the rounded lateral facet, from which it is separated by a sharp medially-arching ridge. The proximal outline is dorsoventrally elongated and broader ventrally than dorsally. Both the dorsal and ventral processes are elongated, the latter slightly further. The bone is about twice longer than lateromedially broad and 1.5 longer than dorsoventrally deep. Its ventral surface bears a marked proximal flexor pit. The distal articulation lacks the lateral condyle, but is not as dorsoventrally deep as the proximal articulation. It has well marked extensor and medial collateral pits, and intercondylar groove, leading proximally to a small extensor pit at the dorsal margin. Based on their resemblance to the former elements, two other phalanges of the sample are identified as a right phalanx 1 (MPCO.V 0060; Fig. 10m–r) and a left phalanx 2 (MPCO.V 0057a; Fig. 10s–x) of the fourth pedal digit. The former is deeper and more lateromedially compressed than that of the articulated pair. It is about 1.5 longer than dorsoventrally deep at its proximal articulation, which is 1.5 deeper than the distal articulation and almost twice deeper than lateromedially broad. That articulation is also lateromedially narrower than the distal and its breadth (lateromedial) is less than half the total length of the bone. It preserves a well-developed process (extensor tuber) at its dorsal portion and a flattened and rugose flexor platform at the ventral margin. A foramen is seen perforating the distal part of the dorsal surface of the bone. The left element shows that the medial distal condyle of the phalanx 2 of digit IV is dorsoventrally deeper, but less distally extensive than the lateral condyle. Those two condyles are also closer to one another at their dorsal part and more set apart ventrally. These pedal digit IV phalanges match the general morphology of those descried for other abelisauroids^2,7,8^. MPCO.V 0006k (Figs Fig. 11y-dd) corresponds to an articulated pair of phalanges 2 and 3 of a left digit IV. Their collateral pits are better developed on the medial side and the lateral distal condyles are somewhat more distally extensive. Both have well-developed extensor and flexor tubers, the latter ventrally flattened as a platform. The medial and lateral condyles are not parallel to one another in distal view.

**Figure 12:**
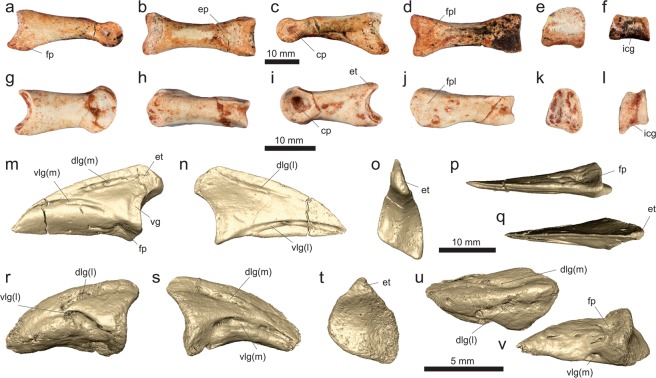
Pedal phalanges of *Vespersaurus paranaensis* gen. et sp. nov. (**a**–**f**) Phalanx 1 of digit III (MPCO.V 0049) in lateral/medial (**a**), dorsal (**b**), lateral/medial (**c**), ventral (**d**), proximal (**e**), and distal (**f**). (**g**–**l**) Phalanx 2 of digit III (MPCO.V 0059) in lateral (**g**), dorsal (**h**), medial (**i**), ventral (**j**), proximal (**k**), and distal (**l**). (**m**–**q**) Ungual phalanx of a right digit IV in medial (**m**), lateral (**n**), proximal (**o**), ventral (**p**), and dorsal (**q**). (**r**–**v**) Ungual phalanx of a left digit I in medial (**r**), lateral (**s**), proximal (**t**), dorsal (**u**), and ventral (**v**). Anatomical abbreviations, as in Fig. 11, plus: dlg(m/l), dorsal longitudinal groove (medial/lateral), icg, intercondylar groove; vg, vertical grove; vlg(m/l), ventral longitudinal groove (medial/lateral).

Other isolated non-ungual phalanges within the sample are dorsoventrally depressed, more elongated, and have a symmetric distal ginglymus. As such, they probably represent digit III phalanges^7^, the two best preserved of which (MPCO.V 0049, 0054) are phalanges 1. MPCO.V 0049 (Fig. 12a–f) is about three times longer than dorsoventrally deep at the proximal articulation, which is nearly twice deeper, but only slightly lateromedially broader than the distal. Both articulations are broader than deep, especially the distal, resulting in an average breadth about 40% the length of the phalanx. They bear shallow, extensor (“D-shaped”) and collateral pits at the distal end, and a subtle intercondylar groove, slightly better developed ventrally than distally. A pair of ventral processes is well developed in the proximal margin, flanking a shallow flexor platform, which is rugose and slightly depressed. The bone is ventrally bent distal to the proximal margin of the extensor pit. Two other isolated phalanges (MPCO.V 0044, 0059) possibly represent the second element of digit III^7^. The best preserved MPCO.V 0059 (Fig. 12g–l) has a trapezoidal proximal outline, deeper than lateromedially broad. A dorsoventrally oriented and laterally arching crest divides the proximal surface in two concave areas, the lateral of which is more dorsoventrally expanded. The proximal articulation is deeper and dorsoventrally broader than the distal, and both are less than half the length of the phalanx. The proximal half of the bone bears well developed medio- and lateroventral corners, the medial of which is more expanded outwards, and both flank a rugose flexor platform. The distal end bears subtle extensor and lateral collateral pits, and a deep medial collateral pit. The intercondylar groove excavates the dorsal, distal, and ventral articular surfaces. The distal outline is deeper than broad, and the lateral condyle is further expanded ventrally than the medial.

In addition to those preserved in the holotype, two isolated pedal ungual phalanges in the sample of *Ves*. *paranaensis* deserve attention. The larger of which (MPCO.V 0022; Fig. 12m–q; see Supporting Information) better fits the proportions of the ungual of the fourth holotypic digit, therefore representing an element from the right side. Its proximal margin is deeper dorsoventrally than half the proximodistal length of the bone. It has a subtriangular proximal outline, with a large subtly convex lateral margin, a straight ventral margin that is the shortest and faces slightly medially, and a medial margin that is concave dorsally and straight ventrally. The dorsal and lateroventral corners formed by those margins extend distally creating the sharp dorsal and ventral margins of the ungual. Those sharp margins give the impression that the bone is extremely compressed lateromedially, but its proximal surface is broader than half its depth. That surface is divided in concave lateral and medial sides by a vertical ridge that extends dorsally from the middle of the ventral margin towards the extensor tuber. This expands proximally, so that the proximal margin of the bone is dorsally convex and ventrally concave in lateral/medial views. In fact, the medial surface is divided into ventral and dorsal parts, set apart by the distal extension of the medioventral corner of the proximal articulation. The dorsal part is dorsoventrally deeper, with the proximal half of its dorsal margin traversed by a groove (dorsal longitudinal groove) that approaches, but does not merges with, the dorsally arching longer ventral longitudinal groove. This starts at the distal tip of the bone, extends proximodorsally along its dorsal margin, and then turns ventrally, occupying about 80% of the length of the ventral margin of the ungual. These represent the abelisauroid “Y-shaped” system of lateral vascular grooves, which is also seen in lateral surface of the second ungual, therefore representing a trait of the “inner” side of the unguals adjacent to the third (main) digit. Clearly, a more typical “Y-shaped” system is seen when the longer ventral groove is more dorsally positioned and connects with the dorsal groove. The proximal subtriangular space between the dorsal and ventral grooves is not protruding, but is proximally bound by a subtle vertical groove (“vg” in Fig. 12m), as in *Mas*. *knopfleri*^2^. Finally, the slightly ventrally facing ventral part of the medial surface represents the distal extension of the ventral margin of the proximal surface, therefore representing the original ventral surface of the bone. Its proximal part is excavated by a “D-shaped” flexor pit, with a flat proximal margin and a rounded, rugose distal margin, as also typical of abelisaurid unguals^6,93^. The lateral surface of the ungual is traversed by dorsal and ventral longitudinal ridges, but these are mostly flanking the respective margins of the bone.

The other isolated ungual phalanx (MPCO.V 0036a; Fig. 12r–v, see Supporting Information) is much smaller, 9 mm of maximal length, and relatively stouter, i.e. less lateromedially compressed, with a proximal dorsoventral depth about 2/3 of the maximal length of the bone. Yet, it has all the typical features of a noasaurid pedal ungual, and seems to represent a left first digit element. Its proximal surface has a slightly concave medial margin and a strongly arched lateral margin, the maximal inflection point of which extends distally along the lateral surface, setting the lateral and ventral margins of the bone apart. The latter bears a flexor pit near the proximal articulation, and the former surface houses the two lateral longitudinal grooves. These are also well-marked on the medial surface of the phalanx, the ventral margin of which forms a sharp ventral keel.

### Results of the phylogenetic analysis

**Figure 13:**
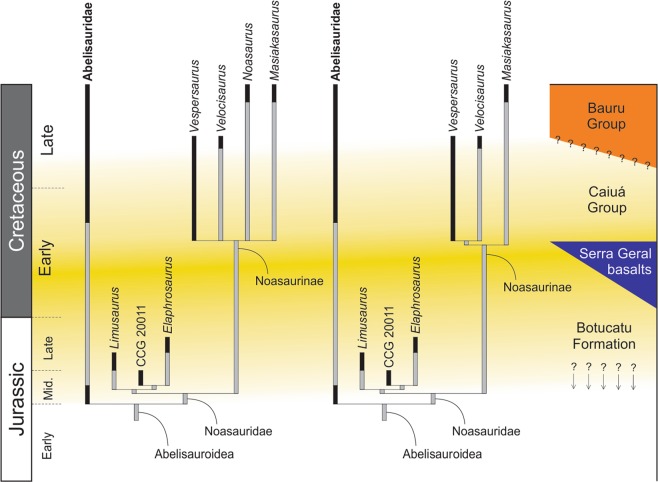
Excerpts (focused on Abelisauroidea) of the Strict Reduced Consensus Trees found in the analyses conducted here (topology to the right excludes *N*. *leali*) calibrated into the Jurassic-Cretaceous timescale and a simplified startigraphic chart of the Paraná Basin. Geological correlations and taxon ranges compiled from various sources^4,21,24,25,27,98^.

The phylogenetic analysis including *De*. *agilis* recovered 17,400 most parsimonious trees (MPTs) of 444 steps, with a consistency index (CI) of 0. 5338 and a retention index (RI) of 0.7380. The optimal results were found in 545 of the 1,000 replications. The strict consensus (SCT) of those MPTs shows *Ves*. *paranaensis* nested within a highly polytomic Ceratosauria (see Supporting Information). The analysis with *De*. *agilis* deactivated recovered 6,612 most parsimonious trees (MPTs) of 438 steps, with a consistency index (CI) of 0. 5411 and a retention index (RI) of 0.7456. The optimal results were found in 893 of the 1,000 replications. The strict consensus (SCT) of those MPTs is congruent with the results of Rauhut and Carrano^4^, and also shows *Ves*. *paranaensis* nested within a highly polytomic Ceratosauria (see Supporting Information). The IterPCR protocol^54^ found that these unresolved relationships were mainly a result of the alternative positions acquired by *Berberosaurus liassicus*, *La*. *indicus*, and MNN tig6 in the recovered MPTs. After pruning these unstable taxa, the strict reduced consensus tree (SRCT) shows the clade formed by *Ceratosaurus* spp. + *Genyodectes serus* as sister to Abelisauroidea, which is composed of Abelisauridae and Noasauridae (see Supporting Information). The nesting of the new species within the latter clade is supported by the presence of middle-cervical vertebrae with postzygapophyses placed caudal to the neural arch pedicle and overhanging the centrum caudally (character 113:0->1), cranial cervical vertebrae with relatively poorly developed (not long and robust) epipophyses (character 115: 1->0/1), and centrum length/height ratio higher than 3 (character 117: 0->1/2). Within noasaurids, *Ves*. *paranaensis* is recovered as a Noasaurinae (Fig. 13) based on the presence of mid-sacral centra with equivalent transverse dimensions relative to other sacral elements (character 131: 1->0), ilium with pubic and ischial peduncles of subequal proximodistal lengths or with a longer ischial peduncle (character 183: 0->1), and metatarsal II with proximal articular surface two-thirds or less the width those of metatarsals III or IV (character 213: 0->1).

The branch supports for Noasauridae are relatively low, with a Bremer support of 2 and absolute and GC bootstrap frequencies of 44% and 34%, respectively. Such supports are similar for Noasaurinae, with a Bremer support of 2 and absolute and GC bootstrap frequencies of 40% and 37%, respectively. The lack of resolution within Noasaurinae results from alternative positions adopted by *N*. *leali* among the MPTs. When this species is pruned from an even more taxonomically restricted SRCT (see Supporting Information), *M*. *knopfleri* is positioned as sister to a clade composed of *Ves*. *paranaensis* and *Vel*. *unicus* (Fig. 13). In this tree, the Bremer support of Noasaurinae is 3 and the absolute and GC bootstrap frequencies are of 43% and 40%, respectively. The clade composed of *Ves*. *paranaensis* and *Vel*. *unicus* is based on a metatarsal IV with a lateromedially compressed shaft with respect to that of metatarsal III (character 220: 0->1). This clade is more poorly supported than Noasaurinae, with a minimal Bremer support and bootstrap frequencies of 37% and 32%, respectively.

Under topologically constrained searches, five additional steps are necessary to place *Ves*. *paranaensis* as sister to all other noasaurids, and eleven and six steps to force a sister-taxon relationship with *E*. *bambergi* and *Li*. *inextricabilis*, respectively. Beyond Noasauridae, nine extra steps place the new species within Abelisauridae (as sister to all other clade members) and 19 steps are required to force its position outside Abelisauroidea (when it is recovered as sister to that clade).

## Discussion

The position of *Ves*. *paranaensis* as a noasaurine abelisauroid is well-supported, but the relationships among noasaurines are unresolved when its four core species are included in the consensus tree. This seems to be mainly a result of the high amount of missing data present in two of the four known species of the clade (*N*. *leali*, 83.2%, and *Vel*. *unicus*, 91.4%). In fact, *Ves*. *paranaensis* (with 64,5% missing entries) represents the first noasaurine with multiple comparable characters with *Mas*. *knopfleri*, the best sampled species of the clade (24.5% missing entries). Indeed, for any resolution to be attained including all these four taxa, it is necessary to identify characters that can be scored (with variation on the states) for at least three of them. Yet, overlapping of skeletal parts among these taxa is minimal, including only metatarsal II for *Vel*. *unicus*, *N*. *leali*, and *Ves*. *parananesis*, that bone plus cervical vertebrae and manual phalanges for *N*. *leali*, *Mas*. *knopfleri*, and *Ves*. *parananesis*, and the tibia (very incomplete in the new species) plus foot for *Vel*. *unicus*, *Ves*. *parananesis*, and *Mas*. *knopfleri*. As such, the discovery of more complete noasaurine remains, especially for the Argentinean taxa, is necessary to substantially help unravel the relationships within the clade.

### Implications for the understanding of Noasaurinae anatomy and evolution

Regardless of the uncertainties on the relations among noasaurines, the larger amount of characters sampled for *Ves*. *parananesis*, at least compared with *Vel*. *unicus* and *N*. *leali*, allows the revision of certain anatomical traits of the group. This is particularly the case for the manus and pes, better preserved in *Ves*. *parananesis* than in any other known noasaurine. The new species shares the generally reduced forearm and manus of abelisauroids. The radius is about half the length of the humerus, proportionally longer than those of carnotaurines^75,78,94^, but close to the condition seen in other abelisauroids such as *Eo*. *mefi*^13^ and *Li*. *inextricabilis*^57^. Metacarpal III is the only metacarpal preserved for *Ves*. *parananesis*. It is stouter than those of *Eo*. *mefi*^13^ and *Li*. *inextricabilis*^57^, and only slightly more elongated than that of *Maj*. *crenatissimus*^75^. In contrast, the non-terminal phalanges of all those abelisauroids (with the possible exception of the first digit I phalanx of *Eo*. *mefi*^13^) are blockier than those of the new species, although the digits of the latter are somewhat reduced compared to those of non-ceratosaur theropods, e.g. *Di*. *wetherilli*^70^. This is also the case for *C*. *nasicornis*^79^ and the isolated manual phalanges referred to *N*. *leali*^84^ and *Mas*. *knopfleri*^3^. Furthermore, the inferred reduction in the number of phalanges of digit III of *Ves*. *parananesis*, as well as the changes to its terminal element, is not seen in *Li*. *inextricabilis*^57^, but resembles the condition of carnotaurines^75^. Hence, the new species shares some modifications towards the reduction of distal forelimb elements seen only in highly nested abelisaurids, whilst retaining plesiomorphic features for Abelisauroidea. This confirms the mosaic pattern of forelimb reduction generally regarded as characteristic of that clade^13,75^.

The foot of *Ves*. *parananesis* has a rather uncommon morphology. The shafts of metatarsals II and IV are strongly lateromedially compressed, so that the central element (metatarsal III) would have endured most of the load during locomotion. Similar conditions are present in other noasaurines, but not to the extreme extent seen in the new species. Indeed, although flattened, the second metatarsal of *N*. *leali*^5^, *Vel*. *unicus*^7^, and *Mas*. *knopfleri*^2^ is not laminar to the same degree as in *Ves*. *parananesis*. In addition, the fourth metatarsal of *Mas*. *knopfleri*^2^ is not transversely compressed, unlike those of *Vel*. *unicus*^7^ and *Ves*. *parananesis*. As for the phalanges, the third ungual of *Ves*. *parananesis* resembles those recovered for *Mas*. *knopfleri*^2^, as well as the fourth ungual of *Vel*. *unicus*^6^, consistent with the condition expected for a weight-bearing phalanx. In contrast, those of digits II and IV are strongly compressed lateromedially. Their inner (facing the central digit) portions, including the condyle and ventral margin, are elevated dorsally relative to their outer parts. Therefore, the ventral margin of the outer portion forms a sharp ventral edge, atypical for a weight-bearing phalanx.

The anatomy of the unguals goes hand in hand with the subtle shortening of pedal digits II and IV of *Ves*. *parananesis*, especially when compared to the general condition of theropds^57,70^. In the articulated pes, they barely extend distally to reach the penultimate phalanx of digit III. A similar digit shortening is seen in *Vel*. *unicus*^6^, and was also reported for a still undescribed noasaurid from Niger^18^. These anatomical traits suggest that *Ves*. *parananesis* could have been a functionally monodactyl animal, with a single central weight-bearing digit, flanked by neighbouring elements that were positioned very close to digit III or perhaps even held free of the ground. As such, we seem to be facing an anatomical adaptation previously unrecorded among archosaurs, although previously hinted-at from footprints from the same geological unit that yielded *Ves*. *parananesis* (see below). Among vertebrates, monodactyl bipedalism is also seen in sthenurine kangaroos^95^, but these differ from *Ves*. *paranaensis* in being saltatorial animals. On the other hand, lateromedially flattened ungual phalanges are also seen in all pedal digits of *Erlikosaurus andrewsi*^96^, an animal that surely employed digits II-IV for weight-bearing. As such, this morphology alone cannot be used as the sole indication to infer that a digit has no function in locomotion. Moreover, the fact that the non-ungual phalanges of *Ves*. *parananesis* are similarly robust in the three central digits also cautions against the off-hand dismissal of a weight-bearing role for digits II and IV. Yet, even if this was the case, the unusual morphology of the unguals suggests they were surely employed in a function other than locomotion. In this context, it is interesting to note that the flexor tubercles of the proximal phalanges in the fourth digit of *Ves*. *parananesis* are significantly more expanded than those of the central (third) digit, as seen in the penultimate phalanx of the hypertensive second pedal digit of dromaeosaurids^97^. Yet, unlike the dromaeosaurid sickle-claw, the pedal unguals II and IV of *Ves*. *parananesis* are not strongly recurved, nor pointed, but have a straight, sharp ventral margin. As such, those elements probably functioned more as slicing/scratching devises (for whatever inferred behaviour) than as a perforating weapon. Also, their extensor tubers are much larger than in the dromaeosaurid ungual II, in a digit that has flexors tubers better developed in its proximal, rather than distal phalanges. Such an arrangement may relate to the contraction of the outer digits via the extension and flexion of their distal and proximal portions respectively, possibly facilitating (again) a monodactyl stance. Finally, the first phalanx of digits II and IV has asymmetrical flexor tubers, more expanded towards their inner side. This suggest that, during retraction, those digits were not only pulled backwards, but also inwards, so as to approach one another behind digit III. Clearly, a more detailed biomechanical study of the *Ves*. *parananesis* foot, which is beyond the scope of this initial contribution, is needed to fully explore its functional implications.

Dating to at least since the earliest Cretaceous, the Botucatu Desert represents one of the largest deserts in Earth’s history, covering an area of about 1.300.000 km^2^ in south-central Brazil and also occupying parts of Argentina, Uruguay, and Paraguay^98,99^. It was home to an assorted fauna, including mammals and dinosaurs, whose presence is inferred based on a huge collection of trackways^100^. Its sand-sheets were later covered by the Valanginian-Aptian Serra Geral basalt flows^24^, also one of the largest in Earth’s history, which covered most of the Paraná Basin. However, the arid conditions continued to prevail after the extrusions (Fig. 13), covering part of the northern half of the Serra Geral basalts in the form of the Caiuá Group^101^. Footprints of very similar morphology to those of the Botucatu Formation are also found in the Caiuá Group^102^, some known from sites near (Fig. 1) the type-locality of *Ves*. *parananesis*^103^, indicating the recurrence of a similar desert-dwelling fauna. Although difficult to substantiate, it is tempting to speculate whether some of those theropod footprints were produced by an animal similar to the new taxon. Indeed, Leonardi^104^ suggested that one such track, found in the same stratigraphic unit at a site about 50 km northwest of Cruzeiro do Oeste, was produced by a functionally monodactyl bipedal “coelurosaur”. This corresponds to three footprints, about 8 cm each in length (stride = 80 cm; nearly 180°pace angulation) and representing, following classical models^105^, a trotting bipedal theropod of less than half a meter hip-height. Despite all the uncertainties involved in the preservation of tracks in aeolian sandstones^106^, these footprints are broadly consistent with the length of the four phalanges of digit III of the holotype of *Ves*. *parananesis*. Comparable monodactyl footprints have also been recorded in the Botucatu Formation^103,104^ and resemble those from the Jurassic La Matilde Formation of Argentina^107–110^, i.e. *Sarmientichnus scagliai*, suggesting that similar dinosaurs may have roamed such arid settings in South America since earlier in the Mesozoic. In fact, it is noteworthy that the gap in the fossil record of noasaurids, from the Late Jurassic to the Early Cretaceous (Fig. 13), generally matches the inferred (even if rather uncertain) age of the Botucatu Formation, and could be explained by the colonisation of arid environments by the group, in which the preservation of body-fossils is not so common. Finally, by identifying a more suitable producer, this cast doubts on recent suggestions, from Chinese tracks^111^, that footprints such as *S*. *scagliai* are better assigned to a didactyl deinonychosaur.

## Supplementary information

Supplementary Information

Supplementary Information

Supplementary Information

Supplementary Information

Supplementary Information

Supplementary Information

Supplementary Information

Supplementary Information
