# Supplementary material for: A new desert-dwelling dinosaur (Theropoda, Noasaurinae) from the Cretaceous of south Brazil
Source: Sci Rep. 2019 Jun 26;9:9379. doi: 10.1038/s41598-019-45306-9 (PMC6594977; doi:10.1038/s41598-019-45306-9)
Supplement: Supplementary file 1 — Supplementary Information [file 41598_2019_45306_MOESM1_ESM.pdf]

A new desert-dwelling dinosaur (Theropoda, Noasaurinae) from the Cretaceous of south Brazil

Max Cardoso Langer, Neurides de Oliveira Martins, Paulo César Manzig, Gabriel de Souza Ferreira, Júlio César de Almeida Marsola, Edison Fortes, Rosana Lima, Lucas Cesar Frediani Sant'ana, Luciano da Silva Vidal, Rosangela Honório da Silva Lorençato and Martín Daniel Ezcurra

Supplementary Information

DETAILS OF THE PHYLOGENETIC ANALYSES

1 - Characters added to the phylogenetic matrix of Rauhut and Carrano (2016):

219) Ischium, proximal end: pubic articular facet dorsoventrally deeper than that for the ilium (0); iliac articular facet dorsoventrally deeper than that for the pubis (1).

220) Metatarsal IV, shaft: uncompressed (0), lateromedially compressed (1) with respect to the shaft of metatarsal III.

2 - Scorings changed from Rauhut and Carrano (2016):

Character 104: changed from (?) to (2) in *Limusaurus inextricabilis* (based on the scorings of Wang et al. 2016 for this taxon).

Characters 106, 107, 115 and 213: changed from (?) to (0) in *Limusaurus inextricabilis* (based on IVPP V15923, MDE pers. obs.).

Characters 109 and 114: changed from (?) to (1) in *Limusaurus inextricabilis* (based on IVPP V15923, MDE pers. obs.).

Characters 154: changed from (1) to (0) in *Limusaurus inextricabilis* (based on IVPP V15923, MDE pers. obs.).

Characters 195, 196 and 204: changed from (?) to (1) in *Velocisaurus unicus* (based on Egli et al. 2016).

Characters 211, 214 and 217: changed from (?) to (1) in *Velocisaurus unicus* (based on MUCPv-41).

Characters 199 and 200: changed from (?) to (1) in *Velocisaurus unicus* (based on Egli et al. 2016).

3 – Data matrix:

Herrerasaurus
0000000000011000000000000000000100100{01}0000000000000000?00000000000
00000000100000000000000000100000000000000000000{01}00010000000001?000000
00?000000000001100?0000000000001000000000000000000000000000000000000-00
0000000000000000

Megapnosaurus
0000010000100000000000000000000001000000000000000000000000000000010000
0000100000000000000000000100000011000010001010211001010000000001001001
0000{01}00000011000000000000000110001011101101000000100010011000000100
010001?000010

Dilophosaurus
00000000?000000??000??0100???00020000000000?00000??000??000?0000?1000?
?????0010000000?1001000?01?0000021100{01}000010101100010011000?0000011

00100{02}0???000001000000100000000100000?000?00010???0?1000?0000000010
00000010000?0010

Allosaurus
00000100000000000000000010000000200001000000000000000100000001110110001
10101001100000010000000001001110202001000001210000010000100000000001000
00000000010000000000100000000-000000{01}0000001000110010000010000102001 1
1011000000110

Abelisaurus
1111120?1?011??101111112101111101110?000101?11111?1001?111?10???10011?
?1???1??1?????????101???
??
??????????

Aucasaurus
11?112??1?0?11???1??11?2??????0????????????110???1????????????????????
????????????????0???????????????????1??????????1???????????{01}?????1?
21111?1?????010?211111111112111001?1??101?11???111???1?0??1?1111??1?11
11??0??1111?0

Berberosaurus
??
?????????????????????????1??????220?000??00???0?{01}1?????????{01}1???
??????????0????????????????????0??11??????????????????????010{01}1???1
0110???????????????

Carnotaurus
111112111?011110110111121011111101111011101110111111111110111???100111
?111?111111111110?10100012?111112211110111012101000111111111111111 0211
?1?10111101002111101111121110011111101111111111?11110?1111????11????1?
????????1?

CCG 20011
??
????????????????????????????????110?0111?0?1??211010?0?0000???????1?0?
??{01}??
?????????????

Ceratosaurus
0000120010000000001000000000101002010000000000000000000000111?11000011
1011100111101001000011111210111122100000000{01}1210000010111100110?1?01
0010020000010000011010100000100111{01}011101??00000?111111010{01}11011
01101111100010???00

Dahalokely
??
????????????????????????1???????2211110101012111000111111{01}?????????
??
?????????????

Deltadromeus
??
???0????
??1????1??111111?10?0?????????????????????????????1?0?01100?1121?111?1
100?0????0

Ekrixinatosaurus
1??1?011??01110?????1110?11?1?1?0????1???11???????????1????1??????????
????????????11??01?0????1???????222?11?001?1???11???????1??{01}???0?0?

??111??0????????????????????????????1?????1????111?????0?011?1110??01?
?1?00????1???

Elaphrosaurus
??
????????????????????????1{01}??????11000111001100211010111100011111?00
101??1?1??1?0111111111010??10?01????1011011000011?0?0100?1101001121010
010010?????00

Eoabelisaurus
?0????????????????0001000000{01}10020101001000?10?01??0??01?011?????00
?1??????????????????00???0?1{12}??????221?110?0101?1010001011111011111
?1021110200??110?0000??101{01}010110111111111011?0{01}00?11?-11???0{01
}{01}101101101111??000111?00

Genusaurus
??
?????????????????????????1???????2??????0???????????0???1???1?????????
?????????????????????????????????11?01??1111????????0?0{01}201??11????
?????????????

Genyodectes
0???0???
???????????000?{01}?0001111???
??
?????????????

Ilokelesia
?1????????????????????????????1110111010?1????????????????????1???????
????????????????????????1???????222110010101210110???1111??????????2??
112?011???
????111???

Indosaurus
??????????????????11?1111?111??????????????????????????????11?????1???
??
??
??????????

Kryptops
1????20111011???
????????????????01100???
??
??????????

Laevisuchus
??
????????????????????????1??????????01?1?0011?011?00?????{01}??????????
???????0??
?????????????

Limusaurus
001?0?0???0?00000?00?000000??000?01{01}???00?0?0?0000??????00?????????
????????1-?????0?0-1???????1??0?000?2000?11???10?21??0??11????????????
10?00?010011011101??100101?11?{01}001110011100{01}10000?00?0????1{12}?
00???1??1????10?1???00

Majungasaurus
1111120111011101011111111011111101100010001110111011111111111111101111
11112111111100110110100012111111221111010101210100010111111{01}1111111

210002101101010021111111?112111001????101111{01}1011?11??11?11??0?1011
?11111100111111?0

Masiakasaurus
00?1?00?111?0?????00000000????00001000100?0?0010?????????????11?1001??
?????10?1110000110011?0?12?0111122101111001110210001111101{01}10111101
111001?0111?01111111111010????????1101111?11010??1110110111210?1111111
1111111110110

Noasaurus
0??1?0011110?0??0???????????
?????????????????10011??01{12}????????01?1?101110??00?????????????????
???????000?????????????????????????0???????????????????0??????????????
?????1???????

Vespersaurus
????????????????????000000??
????????????????????????11?011?1221011{01}1001100210001?1110??101?????
???0?1????1?011?00111001?10????02?10??111???010???1?0?10??????????????
????11111{01}111

Rahiolisaurus
1???
???????????????0??001??????1111100{01}01?0011?12?0?00?????????111?????
?????????????11?????????????????????1??01111010?1?1??0?0?1?11011111?1?
11?0010?????0

Rajasaurus
11????????????????1??1111?111????????????????????1?????????1111??01???
????211????1?0110?101???12????????????????????????011?111??110?11?0???
????0????????????????????????????0???0???110???1???11??111??1?11?1????
?0?0??????

Rugops
111110??1?0111011101000011110??????????????11110011???????????????????
???????????????101101?0???
????0???
??????????

Skorpiovenator
11?112111?01111??????????01????1111110111011011111??11???1????????????
?????1??????111?01???0?0??????????{01}?1???010?21?11??????????????????
2?111?1?11???????????????????????????1?0110011011?????????11??????????
????0?1??1?10

MNN tig6
??
????????????????????????11??????2200101{01}0010112011010111100???111??
???????000??
?????????????

Spinostropheus type
??
?????????????????????????1??????201?01{01}1??01??1?00?????????????????
????????????????0000???????????????????????????????????0?????0010?????
???????0?0???

Velocisaurus
??
??

```
????????????????????????????????????????????????????0?11??0011?1?11111
1111??1??1
```

```
USNM 8415
??????????????????????????????????????????????????????????????????????
??????????????????????????????????????????????????????????????????????
?????????????10001001?????????????????????????????????????????????????
??????????
```

4 – Recovered topologies:

Strict consensus of 17,400 most parsimonious trees (MPTs) resulting from the analysis of the above data matrix, with the *a priori* exclusion of USNM 8415 and *Spinostropheus gautieri*.

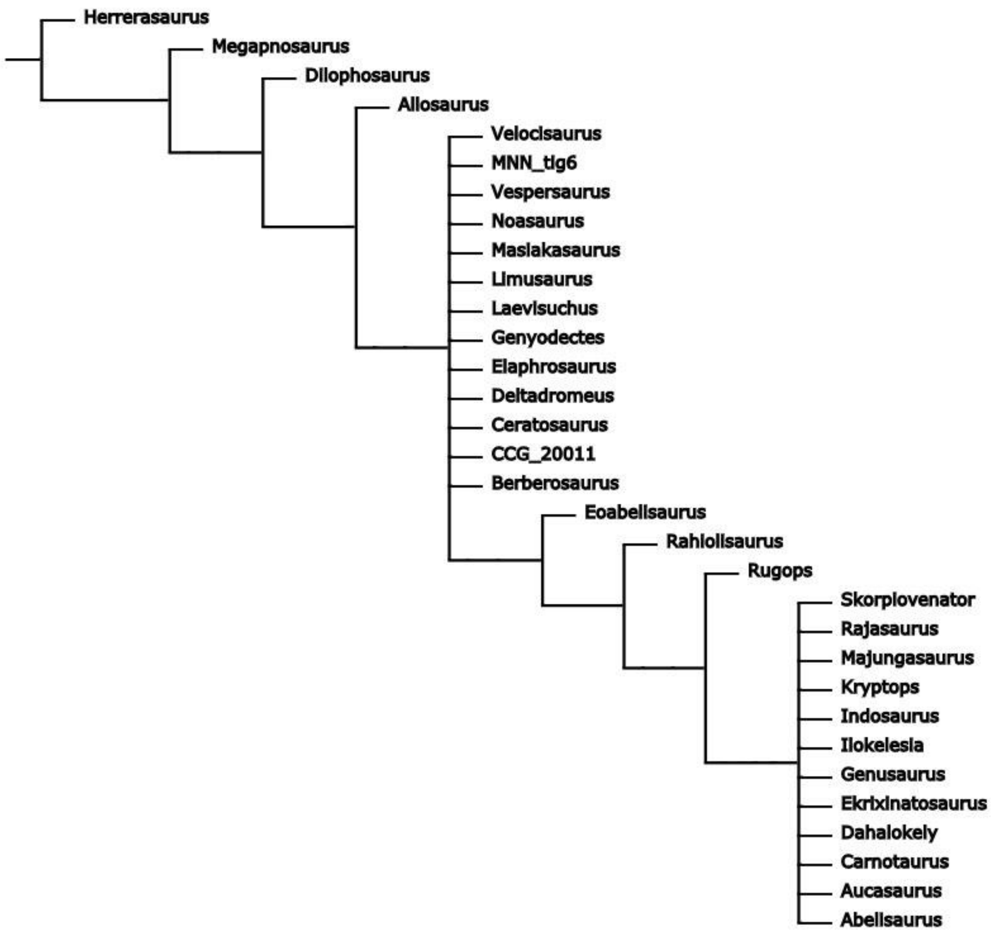

Strict consensus of 6,612 most parsimonious trees (MPTs) resulting from the analysis of the above data matrix, with the *a priori* exclusion of USNM 8415, *Spinostropheus gautieri*, and *Deltadromaeus agilis*.

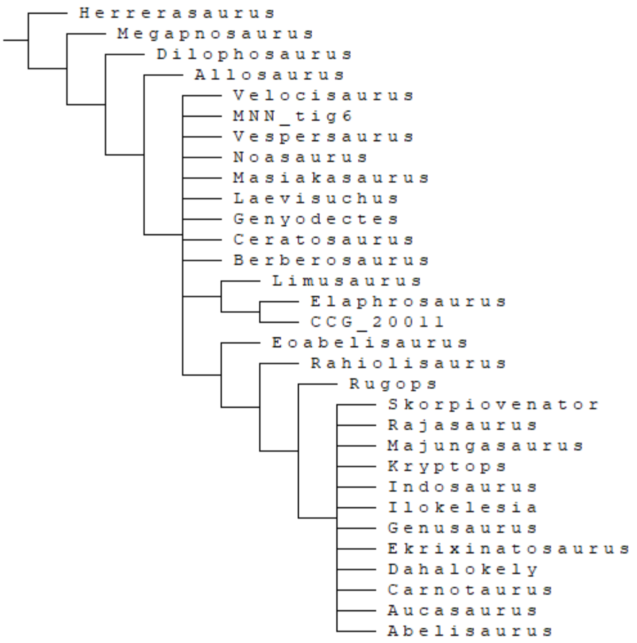

Reduced Strict consensus of 6,612 most parsimonious trees (MPTs) resulting from the analysis of the above data matrix, with the *a priori* exclusion of USNM 8415, *Spinostropheus gautieri*, and *Deltadromaeus agilis*, and the *a posteriori* pruning of *Berberosaurus liassicus*, *Laevisuchus indicus*, and MNN tig6.

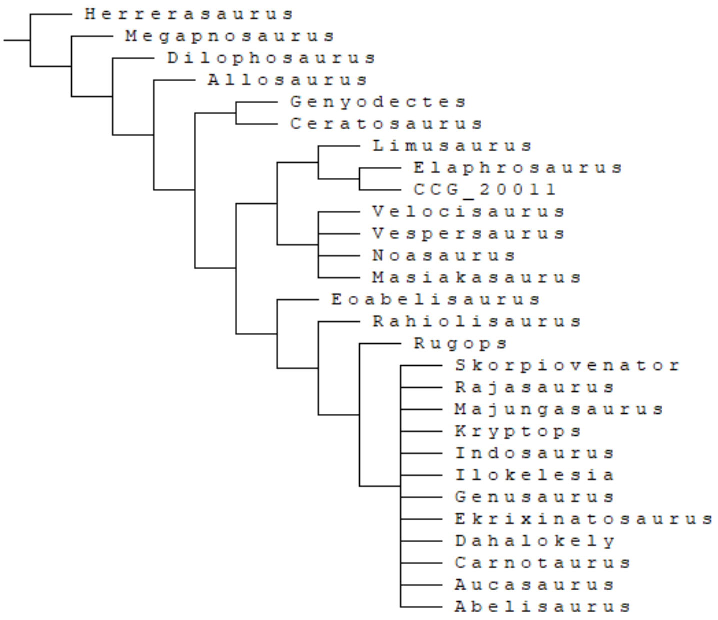

Reduced Strict consensus of 6,612 most parsimonious trees (MPTs) resulting from the analysis of the above data matrix, with the *a priori* exclusion of USNM 8415, *Spinostropheus gautieri*, and *Deltadromaeus agilis*, and the *a posteriori* pruning of *Berberosaurus liassicus*, *Laevisuchus indicus*, MNN tig6, and *Noasaurus leali*.

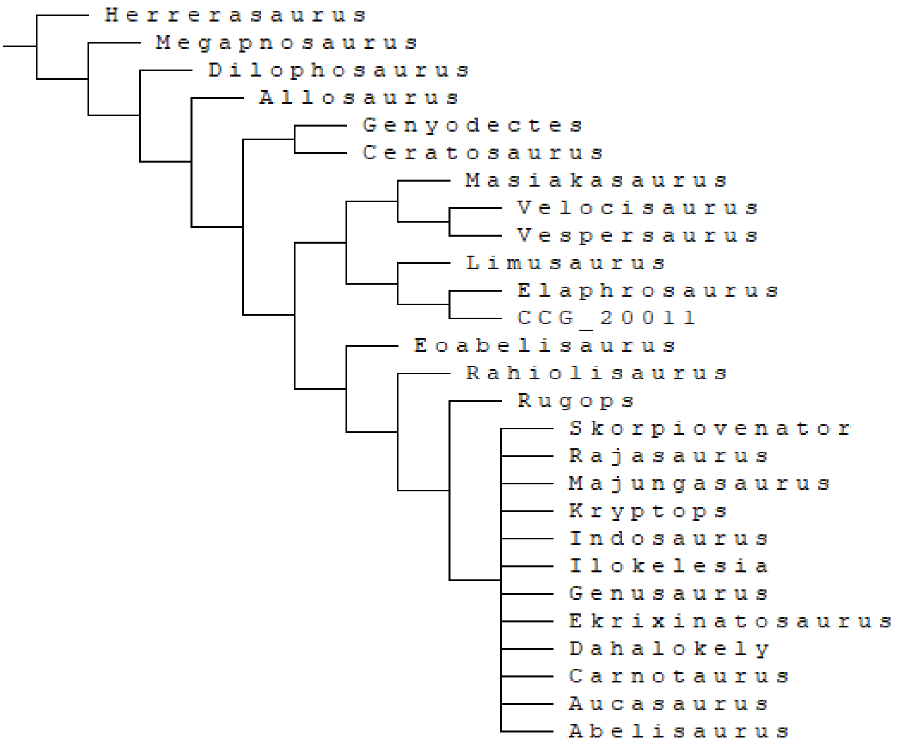

5 – References cited in this Supporting Information

Egli, F. B., Agnolín, F. L., & Novas, F. A new specimen of *Velocisaurus unicus* (Theropoda, Abelisauroidea) from the Paso Córdoba locality (Santonian), Río Negro, Argentina. *Journal of Vertebrate Paleontology* e1119156 (2016).

Rauhut, O. W. M., & Carrano, M. T. The theropod dinosaur *Elaphrosaurus bambergi* Janensch, 1920, from the Late Jurassic of Tendaguru, Tanzania. *Zoological Journal of the Linnean Society* **178**, 546–610 (2016).

Wang, S., Stiegler, J., Amiot, R., Wang, X., Du, G.-H., Clark, J.M., & Xu, X. Extreme Ontogenetic Changes in a Ceratosaurian Theropod. *Cell Biology* **27**, 1–5 (2017).
