# Supplementary figures and images for: A new desert-dwelling dinosaur (Theropoda, Noasaurinae) from the Cretaceous of south Brazil
Source: Sci Rep. 2019 Jun 26;9:9379. doi: 10.1038/s41598-019-45306-9 (PMC6594977; doi:10.1038/s41598-019-45306-9)

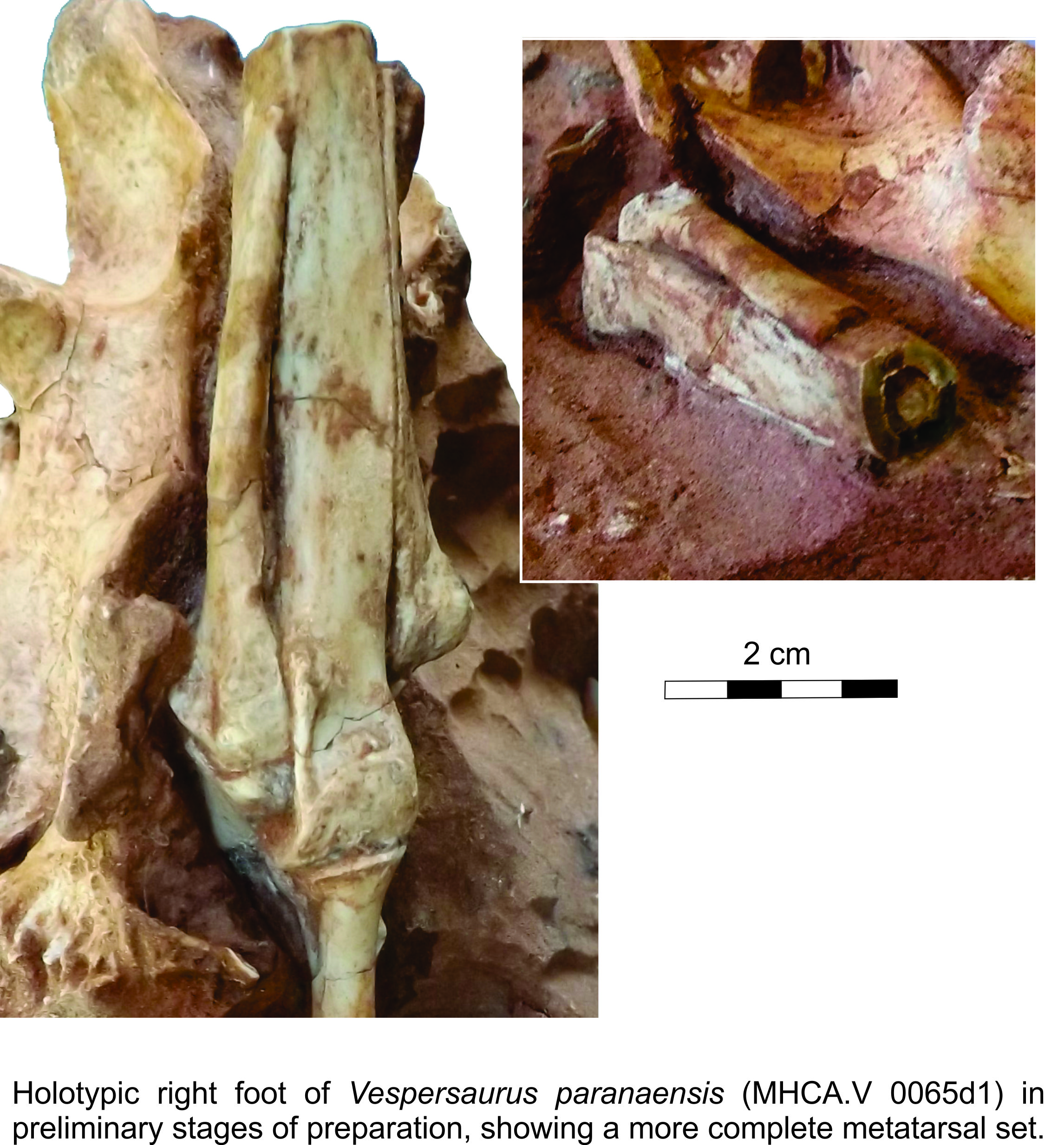

Supplement: Supplementary file 7 — Supplementary Information [file 41598_2019_45306_MOESM7_ESM.jpg]
